# Internet-based vestibular rehabilitation versus written instructions after acute vertigo: A randomised controlled trial

**DOI:** 10.1371/journal.pone.0351092

**Published:** 2026-06-12

**Authors:** Solmaz Surano, Ellen Lindell, Jan Mathé, Hugo Davidsson, Tatjana Tomanovic, Maria Bjurman, Erik Faergemann, Torbjörn Ledin, Anette Rostmark, Helena Grip, Fredrik Öhberg, Gabriel Granåsen, Jonatan Salzer

**Affiliations:** 1 Department of Clinical Sciences, Neurosciences, Umeå University, Umeå, Sweden; 2 Department of Otorhinolaryngology, Head and Neck Surgery, Institute of Clinical Sciences, Sahlgrenska Academy, University of Gothenburg, Gothenburg, Sweden; 3 Department of Clinical Neuroscience, Karolinska Institutet, Stockholm, Sweden; 4 Department of Clinical Science, Intervention and Technology, Karolinska Institutet, Stockholm, Sweden; 5 Department of Biomedical and Clinical Sciences, Linköping University, Linköping, Sweden; 6 Department of Diagnostics and Intervention, Biomedical Engineering and Radiation Physics, Umeå University, Umeå, Sweden; 7 Department of Public Health and Clinical Medicine, Umeå University, Umeå, Sweden; Universiti Malaya Fakulti Perubatan: University of Malaya Faculty of Medicine, MALAYSIA

## Abstract

**Background:**

Acute vestibular syndrome (AVS) causes sudden and continuous vertigo, dizziness, and postural instability and is a common reason for emergency department visits, most commonly due to vestibular neuritis and, in rare cases, stroke. Vestibular rehabilitation (VR) is an evidence-based exercise therapy that facilitates vestibular compensation and is effective in chronic vestibular disorders. However, evidence for VR in acute vertigo remains limited and, despite guideline support, it remains underused in practice. Internet-based VR has demonstrated benefit in chronic dizziness but has not been evaluated in acute vertigo. This study aimed to evaluate the efficacy of an internet-based VR tool compared with standard care (written instructions) in reducing vestibular symptoms after acute onset vertigo.

**Methods and Findings:**

A multicentre, randomised, evaluator-blinded superiority trial was conducted across nine hospitals in Sweden. Adults with ongoing AVS were recruited within 1–7 days after symptom onset and randomised (1:1) to six weeks of internet-based VR with personalised, progressively adjusted home exercises or to written instructions for home-based VR exercises. The primary outcome was the between-group difference in vestibular symptoms at six weeks measured using the Vertigo Symptom Scale Short Form (VSS-SF; range 0–60), with a difference of ≥3 points prespecified as clinically significant. Secondary outcomes included dizziness-related disability, walking speed, and balance. Of 184 randomised participants, 183 were included in the analyses (94 online VR; 89 written instructions; median age 56 years). In the intention-to-treat analysis, both groups improved at six weeks, with no significant between-group difference (adjusted mean difference −2.0 points, 95% CI −4.9 to 0.9; p = 0.18). Per-protocol analyses were consistent (−1.7 points, 95% CI −4.7 to 1.3; p = 0.27). Over 12 weeks, both groups showed reduced vestibular symptoms and improved disability, balance, and walking speed, without significant between-group differences. No serious adverse events were attributed to the interventions, and compliance was high in both groups.

**Conclusions:**

Internet-based VR was not superior to written instructions in reducing vestibular symptoms six weeks after acute onset vertigo. Both groups demonstrated improvement from baseline, with no statistically significant between-group differences. These findings suggest that ensuring access to vestibular rehabilitation exercises may be more important than the specific mode of delivery after acute onset vertigo, and internet-based tools represent viable alternatives for patients who prefer or may benefit from a digital format.

**Trial registration:**

Clinicaltrials.gov NCT05056324. https://clinicaltrials.gov/study/NCT05056324. Registered on September 24, 2021.

## Introduction

Dizziness and vertigo are common reasons for seeking medical attention, accounting for approximately 3% of emergency department visits worldwide [1–5]. Furthermore, an estimated 15% of adults report experiencing dizziness annually [6]. In Sweden, vestibular disorders have been identified as the leading cause of audiovestibular-related sick leave [9,10]. A recent study found that dizziness- and vertigo-related sick leave episodes constituted 0.83% of all sick leave episodes in Sweden from 2008 to 2015 [7]. Consequently, dizziness and vertigo place a considerable burden on healthcare systems through increased use of diagnostic imaging and emergency services, as well as on society through reduced productivity and work absenteeism [8].

Acute vestibular syndrome (AVS) is defined as the sudden onset of dizziness or vertigo, accompanied by nausea or vomiting, head motion intolerance, gait instability, and often nystagmus, persisting for at least 24 hours [9,10]. A common cause of AVS is acute unilateral vestibulopathy, including conditions such as vestibular neuritis or, rarely, a stroke in the posterior circulation [10].

Vestibular rehabilitation (VR) is an evidence-based treatment for chronic dizziness but is implemented in less than 5% of eligible primary care patients [11,12]. A Cochrane review of 39 randomised controlled trials (RCTs) involving 2,441 participants demonstrated moderate-to-strong evidence supporting VR as a safe and effective treatment for peripheral vestibular disorders [13]. Notably, only five of these RCTs specifically investigated populations with acute vestibular syndrome, and these studies were limited by small sample sizes (n = 30–87) [14–18]. Additionally, just one trial employed patient-reported outcome measure (PROM) as the primary outcome measure [18]. PROMs are crucial for capturing patients’ subjective experiences of vestibular symptoms and for evaluating the real-world impact of rehabilitation interventions, particularly when objective measures may not fully reflect treatment outcomes [19].

More recently, VR through supervised group exercise therapy was demonstrated as an effective method for rehabilitating vestibular neuritis, a common cause of acute vertigo [20]. Although effective, supervised group therapy interventions are often limited in availability, not least in rural areas of Sweden, often leaving written instructions as the primary approach.

An internet-based VR tool, available in English and Dutch, has been developed and evaluated for chronic dizziness in primary care settings [11,21]. A study using the Dutch version of the tool showed that patients with chronic dizziness who received the intervention had a mean vertigo symptom scale short form (VSS-SF) score of 7.5 (SD 7.8) six months after symptom onset, compared with 10.9 (SD 9.3) in the control group [21]. A change of ≥3 points on the VSS-SF is considered clinically significant [11,12], and the observed between-group difference of 3.4 in the Dutch trial by van Vugt *et al*., (2019) informed the anticipated effect size of the current trial.

To date, internet-based VR has not been assessed for acute onset vertigo, despite evidence that 30–50% of patients with acute vertigo continue to experience symptoms three months after symptom onset [22]. Prospective longitudinal studies in acute unilateral vestibular loss show that recovery is often heterogeneous. In a randomized controlled trial of acute vestibular neuritis comparing standard care alone (corticosteroid treatment) with standard care combined with intensive vestibular rehabilitation, the standard care group reported higher dizziness-related handicap, measured using dizziness handicap inventory (DHI) scores, and greater overall perceived dizziness at 3- and 12-month follow up, indicating persistent symptoms [20]. A similar pattern was observed in a recent randomized placebo-controlled trial of corticosteroid treatment, in which the placebo group and the low-dose corticosteroid group showed no significant improvement in DHI scores between the 3- and 12-month follow-up. In the latter, all participants were informed about the importance of vestibular exercise, were given verbal and written instructions, and were instructed to initiate the exercises as soon as possible [23]. Together, these findings suggest that although early improvement is common, long-term dizziness remains frequent, underscoring the need to evaluate scalable rehabilitation strategies early after acute onset vertigo.

In this study, we translated and validated an existing online VR tool (*Balance Retraining*, https://balance.lifeguidehealth.org/) and evaluated its effectiveness in a hospital-based cohort.

The primary aim of this study was to assess the online tool’s efficacy, compared with standard care delivered through written instructions, which served as an active comparator, in reducing vestibular symptoms after acute onset vertigo. We hypothesised that patients using the online VR tool, *YrselTräning*, which approximately translates to “vestibular exercise” in Swedish, would achieve significantly faster and greater balance recovery, as measured by the VSS-SF, than those receiving written instructions.

## Methods

We conducted a randomized, controlled, evaluator-blinded, multicentre superiority trial with a 1:1 allocation ratio among adults (≥18 years) with acute vestibular syndrome. The clinical effectiveness of online VR was compared with standard care consisting of written instructions. To ensure equitable access to potential benefits, participants in the standard care arm were granted access to the online VR tool after three months. The study was registered on ClinicalTrials.gov, NCT05056324, prior to enrolling the first patient (https://clinicaltrials.gov/study/NCT05056324), and a detailed protocol has been published [24]. Participant recruitment took place between 1 October 2021 and 1 February 2024, and follow-up was completed on 12 March 2025. This study is reported in accordance with the CONSORT 2025 guideline [25]; see S1 File checklist.

### Participants

Study participants were recruited from nine hospitals in Sweden, including major university and regional hospitals: The University Hospital of Umeå, Sahlgrenska University Hospital, Karolinska University Hospital, Linköping University Hospital, Capio Saint Göran’s Hospital, Sollefteå Hospital, Sunderby Hospital, Sundsvall County Hospital, and Södra Älvsborg Hospital.

Potential participants were identified by the site study coordinator through daily monitoring of emergency department and hospital wards, including internal medicine, neurology, and ear-nose-throat (ENT) wards. This process involved contacting on-call physicians and emergency department staff, manual screening of patient lists, and the use of notice-board advertisements and informational brochures in relevant departments. In addition, ENT outpatient clinics identified potential participants during acute vertigo visits. Procedures for identifying eligible patients were adapted locally by each site principal investigator (PI). The site PI or delegated staff, including physicians, nurses, and physiotherapists, identified potential participants, provided study information, and screened participants using a checklist of inclusion and exclusion criteria.

Participants were eligible if they were aged 18 years or older and presented with acute vestibular syndrome defined as new-onset dizziness or vertigo with a duration of ≥24 hours with pathological spontaneous or gaze-evoked nystagmus, with ongoing symptoms at the time of inclusion. Exclusion criteria included pre-existing vestibular or neurological conditions, inability to use the online VR tool, cognitive or language barriers affecting study comprehension, medical or physical contraindications to the required head movements, and regular use of medications such as anticonvulsants, antiemetics, benzodiazepines, or neuroleptics. Recurring vertigo without a prior diagnosis and transient treatments for current vertigo were permitted. Screening and enrolment occurred within seven days of symptom onset. A full list of inclusion and exclusion criteria is available in the published study protocol [24].

Eligible participants received comprehensive oral and written information about the study, including its purpose, potential risks, and potential benefits. They were informed that participation was voluntary and that they could withdraw at any time without providing a reason. Participants were given the opportunity to ask questions and adequate time to consider participation. Individuals who agreed to participate provided written informed consent with the site principal investigator (PI) or delegated staff prior to any study procedures and received copies of the signed consent form and study information.

### Ethics

The study was conducted in accordance with the Declaration of Helsinki. Ethical approval was granted by the Swedish Ethical Review Authority (Etikprövningsmyndigheten), Uppsala, Sweden, on 7 April 2021 (study ID: CIV-21-05-036744). All participants provided written informed consent prior to participation.

### Interventions

Participants were randomised to the intervention group, receiving a six-week online vestibular training program requiring 15–20 minutes of daily exercises; or the control group, receiving written instructions. Written instructions were chosen as the comparator because they are commonly used as the first-line treatment at most participating sites. Both the internet-based VR tool and the written instructions described head movement exercises consisting of horizontal shaking (“no–no”) and vertical nodding (“yes–yes”) rotations performed in three conditions: eyes open with gaze following the head, eyes closed, and eyes open with gaze fixed. Physiotherapist-led group training sessions were not included due to limited availability and cost-effectiveness concerns. Currently, Sweden has no specific national guidelines for vestibular rehabilitation in acute vestibular syndrome, and no formal decision-support tools are in use.

### Online VR

The internet-based VR tool is a digital platform designed to guide participants through a six-week VR program. As a web application, it is accessible on any internet-connected device, with each participant assigned a personal login. The tool provided participants with customised exercises each week based on their individual progress, assessed through scoring tests. It tracked user progress, sent reminders for exercise sessions, and delivered instructions through text and video. The tool was used exclusively within the clinical investigation and followed the study protocol.

### Written instructions

Participants randomised to the standard study arm received written instructions outlining the six exercises from the online program and were instructed to increase the level of difficulty when possible [26]. They were also provided with general written advice on how to prevent inactivity, secondary dizziness, and fear of movement. The written instructions are similar to a booklet-based vestibular rehabilitation programme developed by Yardley *et al.,* (2012), which has been shown to be an effective stand-alone treatment for chronic vestibular symptoms in primary care. As such, the written-instruction arm represents an active comparator rather than a placebo-like control [27]. Study personnel were specifically instructed to refrain from coaching the participants to ensure that the interventions were tested as is, without any additional support. Data collection followed the same procedure as in the intervention group. After completing the core trial at three months post-randomization, participants in the standard study arm were offered access to the online VR tool and were followed for up to 12 months. The written instructions are provided here, S6 File Vestibular rehabilitation excercises.

### Concomitant and post-trial care

Despite insufficient evidence for long-term benefit, Swedish guidelines recommend a short course of oral corticosteroids for patients with suspected vestibular neuritis. Accordingly, participants fulfilling these criteria were routinely offered prednisolone 50 mg daily for 5 days followed by tapering over the subsequent 5 days [28] Participation in the study did not influence this decision. Other concomitant care was provided according to local practice at each site.

Under the Swedish Patient Injury Act, participants were entitled to compensation should any harm occur. As the study was conducted within the Swedish public health care system, all participants were covered by the Swedish Patient Insurance provided by Landstingens Ömsesidiga Försäkringsbolag (LÖF).

### Outcomes

The measurements were taken at baseline and at three weeks, six weeks, and 12 weeks follow-up.

The primary outcome was the between-groups mean difference of the VSS-SF score at six weeks [29], reported by telephone to an evaluator blinded to study arm assignment. The VSS-SF is a validated and established PROM [30,31] that has been used in several previous VR trials [11,12,21,27,32], and quantifies symptoms through two subscales: VSS-A, capturing autonomic-anxiety symptoms, and VSS-V, capturing vestibular-balance symptoms [29,30,33,34]. The score range is 0–60, and a difference from baseline of ≥3 points indicate a clinically significant change [11,12]. A Swedish translation, and a modified version for acute vertigo (assessing symptoms during the last 24 hours) was developed and validated during the current study and has been previously published [29]. The acute version, used for baseline measurements, was created to reflect the shorter symptom window and fluctuating course of AVS, whereas the standard version, used at follow-up, assessed symptoms over the past month. Both versions demonstrated robust reliability and discriminative ability for assessing vertigo-related symptoms.

Secondary outcomes assessed the intervention’s impact on daily functioning measured through the dizziness handicap inventory (DHI), and mobility measures using a timed 25-foot walk test (T25-FW) and balance tests. The DHI (range 0–100 points: 16–34 mild, 36–52 moderate, and ≥54 severe dizziness) investigates the effects of dizziness on everyday living [35,36]. The DHI has three subscales, each capturing physical, emotional and functional effects of dizziness on everyday living. The timed 25-foot walk test (T25-FW) measures walking speed using a 25-foot course, and the balance test used in this study was performed with the participant standing with both feet, hip-width apart, on a foam balance pad with both arms crossed over the chest and eyes closed while moving the head from side to side at a frequency of 1 Hz.

### Adverse events

Adverse events and serious adverse events were monitored throughout the study and recorded in the electronic case report form (eCRF). Site PIs judged whether events were related to the intervention, and all serious adverse events were reported to the sponsor according to regulatory requirements. Expected transient symptoms such as vertigo, nausea, or mild discomfort during rehabilitation were not classified as adverse events, as these are considered part of recovery, while falls and fractures were recorded separately as predefined study endpoints. Device deficiencies and serious adverse device effects were also captured if they occurred.

### Randomisation and blinding

Participants were randomly allocated (1:1) to internet-based vestibular rehabilitation or written instructions using site-stratified block randomisation with random block sizes of 4, 6, and 8. The allocation sequence was generated in R by the trial statistician (GG) and concealed in the eCRF. Allocation was revealed only after baseline registration.

Because of the nature of the interventions, participants and site staff were not blinded to allocation. Blinded primary outcome assessment was conducted at six weeks by telephone interviewers whose sole role was outcome evaluation.

Several measures were taken to minimise bias: randomisation was stratified by site, with site included as a random effect in the primary analysis to account for potential bias from differences in patient populations. Study staff were instructed to focus on data collection rather than coaching, thereby isolating the rehabilitation effect to the assigned intervention. The trial statistician remained blinded to allocation until the analyses had been completed.

The randomisation and blinding procedure is described in detail in the study protocol [24].

### Sample size

Initially, a sample size of 320 participants was targeted, based on a previous three-armed randomised controlled trial of the Dutch version of the tool conducted in a primary care setting by van Vugt *et al*., (2019) where a between-group difference of 3.4 points in VSS-SF scores (7.5 online VR vs 10.9 usual care) was observed [21]. The initial sample size estimation assumed 90% power, an alpha level of 5%, and a 15% attrition rate, without accounting for correlations between pre- and post-intervention measurements.

Due to slower-than-anticipated recruitment, inclusion in the study was halted after 184 participants had been randomised, and an updated power calculation was performed. This calculation incorporated an expected correlation of 0.6 between pre- and post-intervention measurements, as outlined by Shieh *et al*., (2020) and implemented using the Superpower package [37]. A common standard deviation of 8.7 points was assumed, corresponding to the pooled standard deviations reported by van Vugt *et al*., (7.8 online VR and 9.3 points usual care). Under these assumptions, a total sample size of 184 participants would be sufficient to detect a difference of 3.4 points with 90% power at an alpha level of 5%. No attrition was incorporated in the updated power calculation as multiple imputation was used to handle missing data.

### Statistical methods

Baseline characteristics in both arms were described using descriptive statistics. The primary outcome was analysed on the intention to treat (ITT) cohort with missing data imputed using multiple imputations, performed using multivariate imputation by chained equations (MICE), implemented via the mice package (version 3.17.0). Ten imputation datasets were generated: each derived from 10 iterations. Variables included in the imputation model were baseline characteristics (as detailed in Table 1) and the outcome measures (VSS-SF, DHI, and T25-FW) at baseline and at 6 weeks. For scales composed of ranked items, imputation was performed at the individual item level.

**Table 1:** Baseline characteristics of participants allocated to either online vestibular rehabilitation or written instructions. Data are presented as numbers (percentages) unless otherwise specified.

Characteristics	Online VR (n = 94)	Written instructions (n = 89)	Total sample (n = 183)
Female	51 (54.3)	40 (44.9)	91 (49.7)
Age, median (IQR)	56.5 (46.0–67.0)	53.0 (41.0–63.0)	56.0 (44.0–66.0)
BMI, median (IQR)	26.2 (24.2–30.8)	25.6 (23.7–28.6)	25.9 (23.8–29.4)
Level of education^†^			
< 9 years	6 (6.5)	1 (1.1)	7 (3.9)
Primary school (9 years)	10 (10.8)	10 (11.4)	20 (11.0)
High school (12 years)	34 (36.6)	29 (33.0)	63 (34.8)
College/University (>12 years)	43 (46.2)	48 (54.5)	91 (50.3)
Baseline diagnosis			
Vestibular neuritis (VN)	86 (91.5)	78 (87.6)	164 (89.6)
Vertigo not otherwise specified	3 (3.2)	5 (5.6)	8 (4.4)
Stroke^††^	3 (3.2)	3 (3.3)	6 (3.3)
Acute vertigo with sudden sensorineural hearing loss	1 (1.1)	3 (3.3)	4 (2.2)
Zoster oticus	1 (1.1)	0 (0.0)	1 (0.5)
Corticosteroid treatment*	68 (79.1)	57 (73.1)	125 (76.2)
No. of chronic diseases**			
0	58 (61.7)	65 (73.0)	123 (67.2)
1	23 (24.5)	14 (15.7)	37 (20.2)
2	10 (10.6)	9 (10.1)	19 (10.4)
≥3	3 (3.2)	1 (1.1)	4 (2.2)
No. of daily medications			
0	26 (27.7)	33 (37.1)	59 (32.2)
1	18 (19.1)	21 (23.6)	39 (21.3)
2	9 (9.6)	14 (15.7)	23 (12.6)
≥3	41 (43.6)	21 (23.6)	62 (33.9)
Psychiatric disorders	7 (7.4)	9 (10.1)	16 (8.7)
Depression	2 (2.1)	3 (3.3)	5 (2.7)
Anxiety disorders	1 (1.1)	3 (3.3)	4 (2.2)
Exhaustion disorder/burnout depression	2 (2.1)	3 (3.3)	5 (2.7)
Other^†††^	2 (2.1)	2 (2.2)	4 (2.2)
Use of hearing aid	9 (9.6)	6 (6.7)	15 (8.2)
VSS-SF score, mean (SD)	19.4 (10.3)	20.1 (10.2)	19.7 (10.2)

* Corticosteroid treatment following VN diagnosis.

**Chronic diseases: non-specific lung disease, cardiac disease, peripheral arterial disease, stroke, diabetes mellitus, muscle-, skeletal-, or joint disease, and cancer.

†Data on this variable was missing for two participants: online vestibular rehabilitation (n = 1), written instructions (n = 1).

†† Stroke was diagnosed with an initial CT scan and confirmed with an MRI.

††† Attention deficit disorder, schizoaffective psychosis

The primary outcome was analysed using analysis of covariance (ANCOVA), with adjustment for baseline outcome values, in accordance with standard practice in randomised controlled trials [38], as well as for other prespecified potential confounders: diagnostic group (categorical), sex (categorical), and age (continuous). A random effect for site, accounting for stratified randomisation, was also included in the model.

Both the primary outcome and the secondary outcomes were longitudinally evaluated using linear mixed models (LMM). The analysis was conducted on the per-protocol cohort and the number of participants at each time point is reported. The LMM was used to analyse outcomes as a function of time (four time points: baseline, 3, 6, and 12 weeks) and intervention group, with random effects for subject and site. All longitudinal models included fixed terms for sex, body mass index (BMI), diagnostic group (vestibular neuritis, stroke, or other) and age at baseline. A random intercept was specified for the sites, with an autoregressive covariance structure of order 1 (AR1) applied to account for correlations between repeated measures within participants at each site. Estimated marginal means were calculated for each intervention group at all time points. Group differences were assessed through post hoc pairwise comparisons using treatment-versus-control contrasts. The marginal means were evaluated at the sample mean for age and BMI. P-values were adjusted using the least significant difference (LSD) method. Results from the longitudinal analyses are presented using estimated marginal means per intervention group as well as the estimated difference between intervention groups at each time point. For T25-FW, analyses were performed on the logarithmic scale due to skewness. Results are presented as ratios of estimated marginal geometric means.

A *p*-value <0.05 was considered to indicate statistical significance. Statistical analyses were conducted using SPSS 29 (IBM Corp., Armonk, NY, 2023) and R 4.4.3 (R Core Team, Vienna, Austria, 2023).

### Patient and public involvement

Patients were not directly involved in the design of the present trial or in translating the online VR tool, which was adapted from the existing *Balance Retraining* programme (https://balance.lifeguidehealth.org/). As part of our previous validation study of the Swedish Vertigo Symptom Scale – Short Form (VSS-SF), however, pilot testing was conducted with four patients admitted for acute vertigo to ensure comprehensibility and usability of the instrument [29]. No patients were involved in interpreting the results or in writing the manuscript. A plain language summary of the study findings will be disseminated to all participants, and results will be shared with relevant patient communities.

## Results

**Fig 1:**
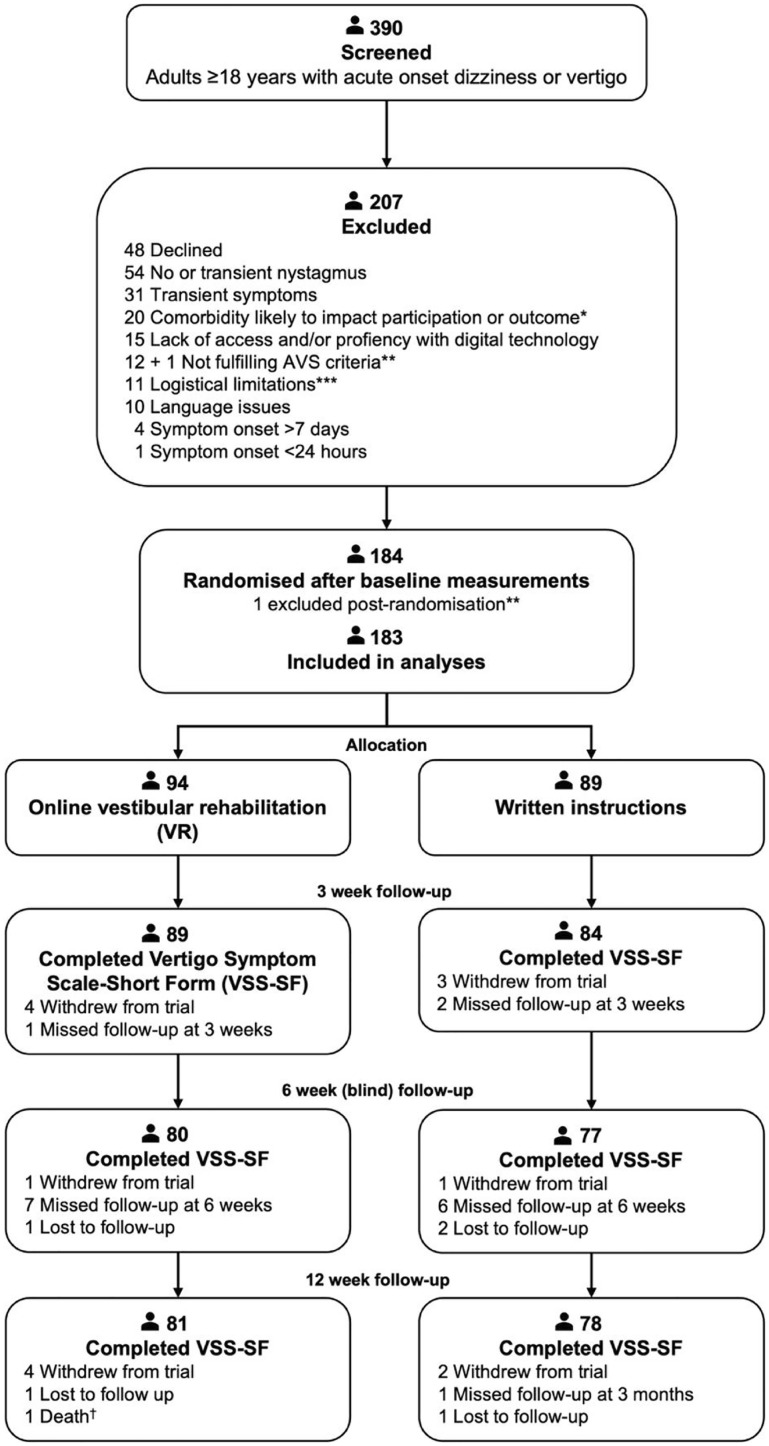
CONSORT 2025 flow diagram. **Flow diagram of the progress through the phases of the trial.** *Comorbidities leading to exclusion included pre-existing vestibular disease, severe cognitive difficulties, recent fracture or injury, inability or risk associated with performing head movements. **Thirteen participants did not meet AVS criteria: nine were diagnosed with BPPV, and four with suspected Ménière’s disease. Of these, 1 person was excluded post-randomization (written instructions group) due to not fulfilling the AVS criteria for nystagmus. ***Exclusions due to staff unavailability or equipment malfunctioning at baseline tests. † Participant had withdrawn from the trial prior to the follow-up.

Participants were recruited between 1 October 2021 and 1 February 2024 from nine Swedish hospitals. The flow of the participants through the trial, including screening, reasons for exclusion, and numbers with complete data for the primary outcome, is shown in Fig 1. A total of 390 individuals were screened for inclusion, and 184 were initially randomised, with 94 in the online vestibular rehabilitation group and 90 in the written instructions group. One participant, randomised to written instructions, was subsequently found not to meet the inclusion criteria and was excluded, leaving 183 participants included in the analyses. Baseline characteristics were similar between groups, with 50% of participants being female, a median age of 56 years, and 90% having a baseline diagnosis of vestibular neuritis, Table 1.

In the online vestibular rehabilitation group, 89 of 94 participants (95%) completed the VSS-SF at 3 weeks, 80 (85%) at 6 weeks, and 81 (86%) at 12 weeks. In the written instructions group, 84 of 89 participants (94%) completed the VSS-SF at 3 weeks, 77 (87%) at 6 weeks, and 78 (88%) at 12 weeks, Fig 1.

### Primary outcome

In the ITT analysis, Table 2, baseline VSS-SF scores were similar between groups, with a mean of 19.2 (SD 10.2) in the online VR group and 19.9 (10.2) in the written instructions group. At six weeks, both groups showed improvement, with mean scores decreasing to 11.1 (8.9) and 13.1 (10.5), respectively. After adjustment for baseline symptom severity and other prespecified covariates, the adjusted mean VSS-SF scores at six weeks were 12.2 (95% confidence interval 8.4 to 15.9) in the online VR group and 14.1 (10.1 to 18.1) in the written instructions group. The adjusted mean difference was −2.0 points (−4.9 to 0.9; *p* = 0.18).

**Table 2:** Comparison of primary outcome between treatment groups.

	Mean score (SD)		Adjusted mean (95% CI)*		Adjusted difference (95% CI)**	
Primary outcome measure	Online VR	Written instructions	Online VR	Written instructions	Online VR vs Written instructions	*p*-value
**VSS-SF (ITT)†**						
Baseline	19.2 (10.2)	19.9 (10.2)	–	–	–	–
6 weeks	11.1 (8.9)	13.1 (10.5)	12.2 (8.4–15.9)	14.1 (10.1–18.1)	−2.0 (−4.9–0.9)	0.18
**VSS-SF (per protocol)††**						
Baseline	19.4 (10.3)	20.1 (10.2)	–	–	–	–
6 weeks	10.9 (9.3)	12.8 (10.9)	11.8 (7.9–15.8)	13.5 (9.5–17.5)	−1.7 (−4.7–1.3)	0.27

SD = standard deviation; CI = confidence interval; VR = vestibular rehabilitation; VSS-SF = Vertigo Symptom Scale – Short Form (range 0–60); clinically relevant difference = 3 points.

*Estimated marginal means from analysis of covariance (ANCOVA), adjusted for baseline score, age, sex, diagnostic category, baseline symptom intensity, and study site.

** Adjusted between-group difference based on estimated marginal means. Negative values in the adjusted difference indicate greater improvement in the online VR group.

† Intention-to-treat analysis included all randomised participants, with 3 weeks carry forward (n = 2) and multiple imputation (n = 24) for missing data, n = 94 in online VR group; n = 89 in written instructions group.

†† Per-protocol analysis included only participants who completed all VSS-SF items at six-week follow-up (n = 80 in online VR group; n = 77 in written instructions group).

In the per-protocol analysis, Table 2, results were consistent with the ITT cohort. At six weeks, adjusted mean scores were 11.8 (95% confidence interval 7.9 to 15.8) in the online VR group and 13.5 (9.5 to 17.5) in the written instructions group, with an adjusted mean difference of −1.7 points (−4.7 to 1.3; *p* = 0.27). The study was powered to detect a between-group difference of 3.4 points based on prior evidence; however, the observed difference was smaller than both the anticipated effect size and the threshold for clinical relevance of 3 points.

**Fig 2:**
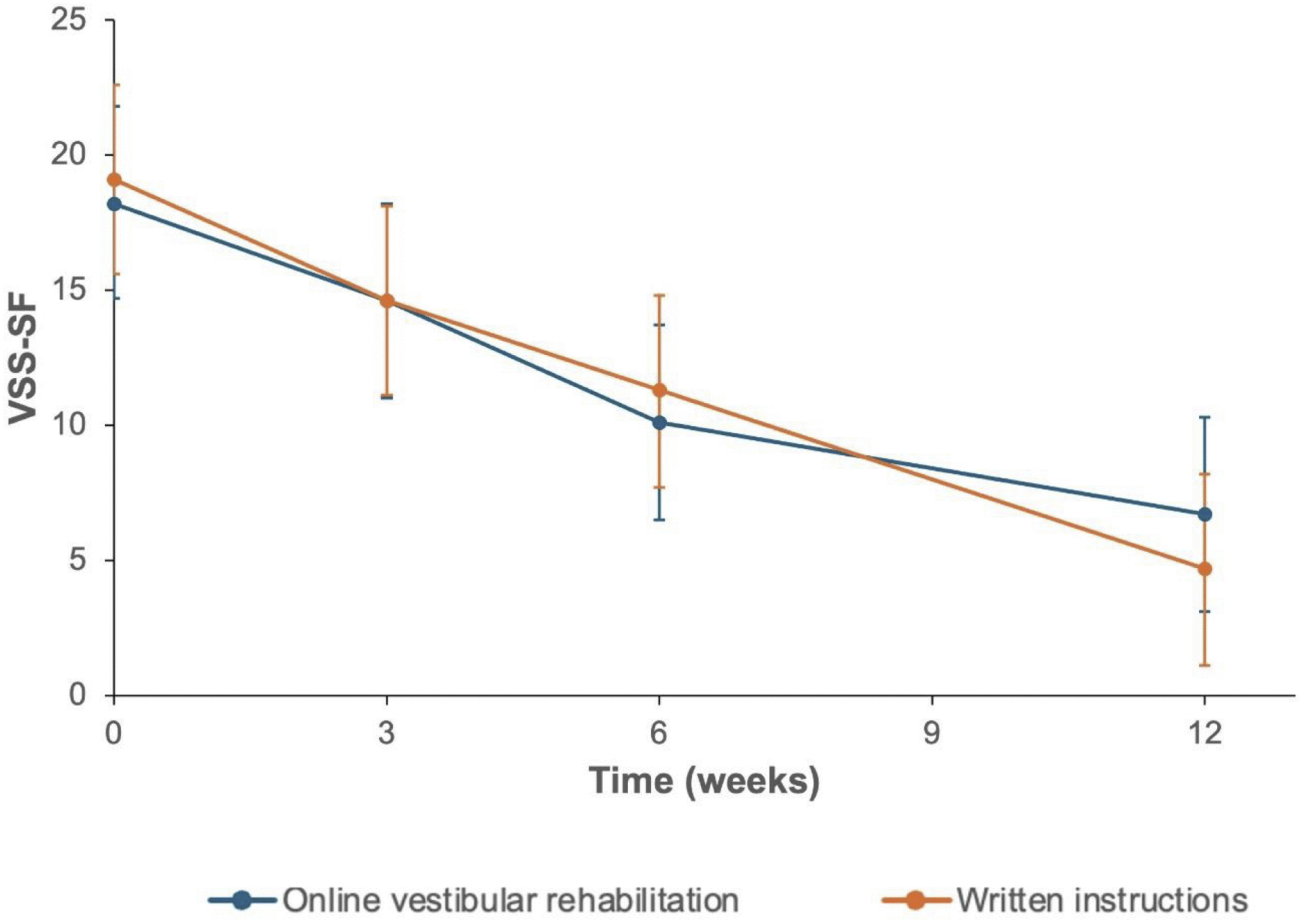
VSS-SF scores over time by treatment group. Estimated marginal means from linear mixed models for VSS-SF (vertigo symptom scale short form) score at baseline, 3, 6, and 12 weeks. Analyses were based on the per-protocol cohort. A total of 81 participants in the online vestibular rehabilitation group and 78 in the written instructions group completed the 12-week follow-up. Error bars represent 95% confidence intervals.

Longitudinal analysis using linear mixed models demonstrated progressive improvement in VSS-SF scores over the 12-week period in both groups, Fig 2. At 3 and 6 weeks, differences between groups were small and non-significant. At 12 weeks, adjusted mean VSS-SF scores were 6.7 in the online VR group and 4.7 in the written instructions group (adjusted difference 2.1, 95% confidence interval −0.8 to 5.0; *p* = 0.16). A similar pattern was seen observed for the VSS-SF subscale scores. The online VR group scored slightly higher on the VSS-A subscale at 12 weeks (adjusted difference 1.3, 0.1 to 2.6; *p* = 0.03) at 12 weeks, although this did not meet the threshold for clinical relevance, S2 File (S2.1 Table).

### Secondary outcomes

The dizziness handicap inventory (DHI) total and subscale scores declined from baseline to 6 and 12 weeks in both groups S2 File (S2.1 Fig). At 12 weeks, adjusted mean scores were 14.9 in the online VR group and 14.8 in the written instructions group (adjusted difference 0.2, 95% confidence interval −6.2 to 6.5). No significant between-group differences were observed at any timepoint for the DHI or its subscales, S2 File (S2.2 Table).

Functional measures also showed consistent improvements from baseline to 12 weeks. The adjusted mean time maintaining balance on a foam pad increased from 6.4 to 19.5 seconds in the online VR group; and from 6.7 to 18.6 seconds in the written instructions group, and there were no significant between-group differences at any time point, S2 File (S2.2 Fig, S2.3 table). Similarly, walking speed improved from baseline to 12 weeks, as reflected by a decrease in the median time to complete the 25-foot walk test from 8.6 seconds to 6.4 seconds in the online VR group; and from 7.8 seconds to 5.7 seconds in the written instructions group. There were no significant between-group differences at any timepoint, S2 File (S2.4 Table).

In the predefined subgroup analyses, no subgroup demonstrated a statistically significant treatment effect, S3 Fig Subgroup analyses. However, there was a significant interaction by comorbidity status (*p* = 0.006), with numerically greater improvement observed in the online VR group among participants with comorbidities (post-hoc).

**Fig 3:**
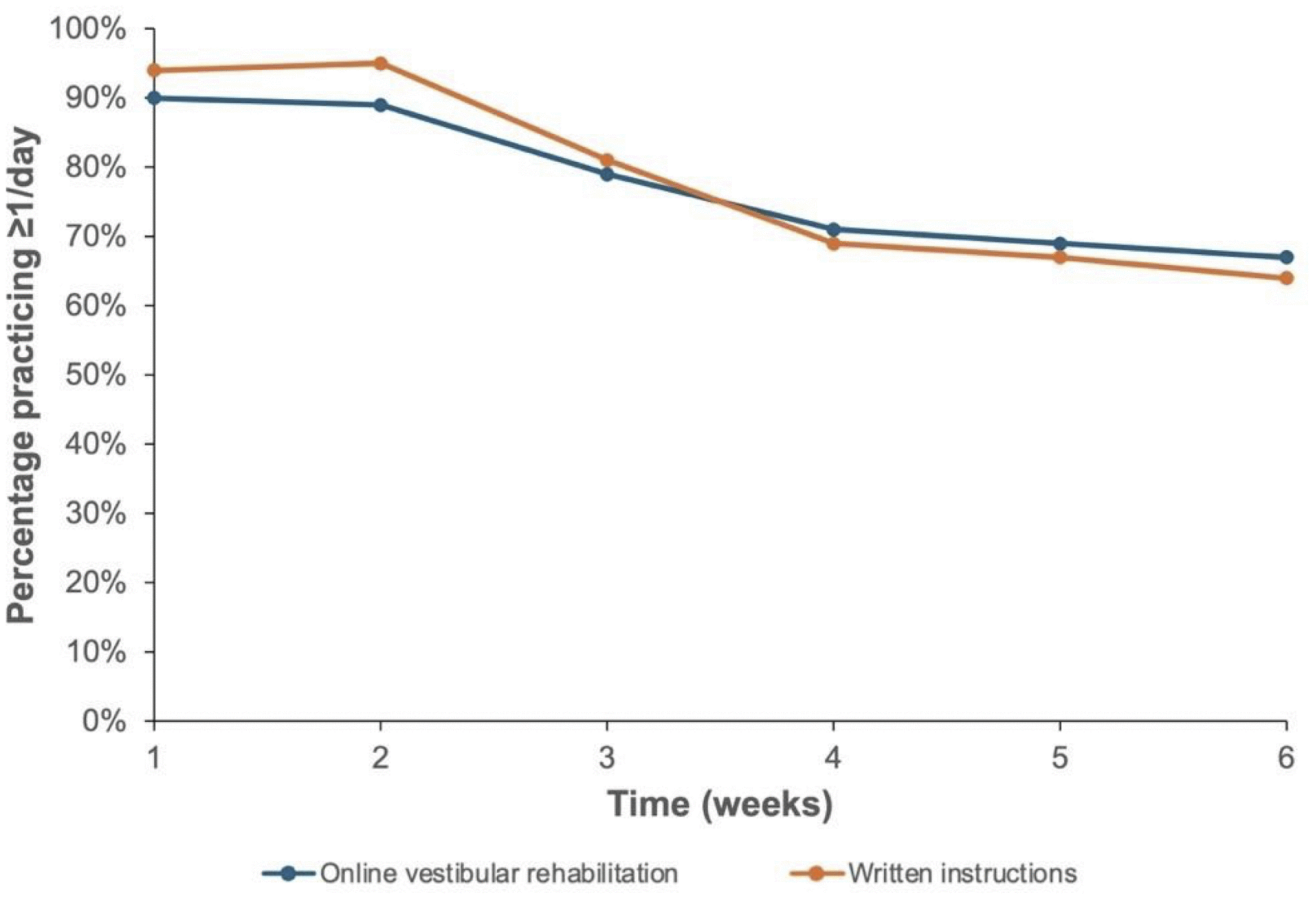
Exercise compliance over the 6-week intervention period. Percentages of participants in each group who reported performing vestibular exercises at least once per day, recorded weekly during the 6-week intervention.

Compliance was high in both groups. Using a definition of compliance as performing the exercises at least once daily, 89–95% of participants were compliant during weeks 1–2; 69–81% during weeks 3–4; and 64–69% during weeks 5–6 (Fig 3). No significant differences in compliance were observed between the study arms.

### Adverse events

A total of 21 serious adverse events were reported during the trial, including one death from myocardial infarction in the online vestibular rehabilitation group and one intensive care unit admission due to an unspecified cardiac event in the written instructions group. Ten participants required non-intensive care hospitalization, three in the written instructions group and seven in the online vestibular rehabilitation group. One case of recurrent vestibular neuritis occurred in the online rehabilitation group. Importantly, none of the serious adverse events were considered related to the vestibular exercises in either group.

In total, 158 non-serious adverse events were documented, with each distinct symptom recorded as a separate event. The most common non-serious adverse events included symptoms of the common cold, headaches, migraine episodes, COVID-19, influenza, and falls without fractures. Less frequently reported were tonsillitis, neck or back pain, palpitations, epistaxis, and gastroenteritis.

Two adverse events were considered likely related to the exercises: one participant in the online group reported transient arm numbness and discontinued the exercises; another in the written instructions group experienced neck and shoulder pain, also leading to exercise discontinuation. Both participants remained in the study and completed follow-up assessments.

## Discussion

In this multicentre randomised controlled trial, no differences were found in vestibular symptom reduction between internet-based vestibular rehabilitation (VR) and standardised written instructions among patients with acute vestibular syndrome (AVS). Both groups showed clinically meaningful within-group improvements on the vertigo symptom scale short form (VSS-SF), with mean reductions of approximately 7–8 points at six weeks. The adjusted between-group difference did not reach statistical significance and was below the predefined threshold for clinical relevance of 3 points [12].

All secondary outcomes, including the dizziness handicap inventory (DHI) and functional mobility measures, also improved over time in both groups without significant differences between them. The mean DHI scores at 6 and 12 weeks were low, corresponding to minimal or no disability due to dizziness, suggesting that both groups reached satisfactory rehabilitation outcomes. Compliance with the prescribed exercises was high in both arms throughout the six-week intervention period, and no serious adverse events related to the exercises were reported.

Symptom improvement over time was observed in both groups in both the intention-to-treat and per-protocol analyses. The similarity of results across these analytic strategies strengthens the conclusion that neither delivery method was superior. In the per-protocol population, which included participants who adhered fully to the assigned intervention, the online VR group showed a numerically greater reduction in symptoms, with an adjusted between-group difference of 1.7 points on the VSS-SF at six weeks. However, this difference was neither statistically significant (*p* = 0.27) nor clinically meaningful, as it did not meet the predefined threshold of 3 points on the VSS-SF scale [11,12]. Overall, the consistency between analyses supports the robustness of the primary findings.

These findings suggest that ensuring access to vestibular rehabilitation exercises may be more important than the specific mode of delivery after acute onset vertigo. Accordingly, personal preferences and contextual factors could guide the choice between digital and paper-based formats in clinical practice.

Our findings differ from those reported in studies of patients with chronic vestibular symptoms. In the trial by van Vugt *et al*., (2019) internet-based VR, with or without physiotherapy support, was associated with a significant and clinically relevant reduction in vestibular symptoms compared with usual care in older adults [21]. That study showed between-group differences on the VSS-SF exceeding 4 points at six months. This discrepancy may reflect differences in timing and natural history. Patients with AVS often experience spontaneous recovery over time [10], limiting the potential margin for added benefit from an online intervention. On the other hand, up to 50% of patients may be affected by chronic dizziness after acute vertigo according to an earlier study, suggesting that both study arms in our study benefited from the interventions [22]. In contrast, patients with chronic symptoms may have more to gain from structured or guided programs. Moreover, our control group received an active intervention in the form of standardised written instructions, whereas previous VR trials have often used “usual care” or minimal intervention as the comparator [11,21]. Given the strong evidence supporting VR for peripheral vestibular disorders [13], it would not be ethically appropriate to withhold vestibular rehabilitation exercises entirely. Instead, our study compared a commonly used delivery format (written instructions) to the online intervention, offering a more pragmatic evaluation of digital implementation in acute care settings. The use of an active comparator may have reduced the likelihood of detecting superiority but increases the clinical relevance of our findings.

The relatively favourable outcomes observed in the written instruction group likely reflect several factors. First, the comparator consisted of structured vestibular rehabilitation exercises rather than passive advice, resembling booklet-based vestibular rehabilitation exercises previously shown to be effective as a stand-alone treatment for chronic dizziness in primary care [27]. Second, participants were enrolled early after symptom onset, which may have enhanced early vestibular compensation. Finally, adherence to the prescribed exercises was high in both groups, which may exceed what is typically achieved under routine clinical conditions. Together, these factors may explain why the active comparator group achieved better outcomes than would be expected with usual care.

Although no subgroups demonstrated a statistically significant between-group treatment effect, exploratory analyses suggested a potential interaction with comorbidity status. Participants with one or more comorbidities tended to benefit more from online VR than those without comorbidities. Similarly, visual trends suggested that younger participants, individuals with more severe symptoms at baseline, and those who were randomised later after symptom onset, may derive greater benefit from the digital format, S3 Fig Subgroup analyses. These patterns, while not conclusive, may guide future hypothesis-driven research and patient selection for digital interventions.

In addition, at 12 weeks, a statistically significant albeit small between-group difference was observed on the VSS-A (autonomic-anxiety) subscale, favouring the written instructions group. However, this difference did not reach the threshold for clinical relevance of 3 points. The result should be interpreted with caution, as it may represent a chance finding due to multiple comparisons, and there was no consistent pattern of anxiety-related benefit in either group across other time points.

### Strengths and limitations of this study

Several strengths enhance the validity of our findings, including the multicentre design, evaluator blinding, and high follow-up rates. The use of both intention-to-treat and per-protocol analyses provides robustness, and multiple imputation addressed missing data. Furthermore, the inclusion of PROMs at multiple time points strengthens the clinical relevance of the results, particularly in a population where objective measures may not fully capture patient experience [19].

Some limitations must be acknowledged. The anticipated effect size was derived from a trial conducted in patients with chronic vestibular disorders in primary care settings [21]. Although this estimate was empirically grounded, treatment effects in AVS may be smaller than those observed in chronic dizziness populations, which could partly explain the lack of between-group differences observed in the present trial.

Compliance may also have been elevated by participation in a trial context, potentially reducing observable differences between groups. Booklet-based vestibular rehabilitation without supervision has been associated with self-reported adherence rates of approximately 34–44% in patients with chronic dizziness, with higher exercise intensity but not significantly higher adherence when telephone support is added [27]. In another primary care cohort with chronic dizziness, 71% of participants reported performing VR home exercise programs most days, although only 55% continued for at least nine weeks or until symptoms subsided, indicating attrition over time [12]. In a randomized trial of patients with unilateral peripheral vestibular dysfunction, adherence rates for home exercise were high (~77%) in both the conventional exercise group and in the virtual reality group, with both groups maintaining an exercise diary [39].

Against this background, the high adherence observed in both study arms of the present trial may reflect the provision of structured instructions and regular follow-up assessments within a clinical trial setting, rather than adherence levels typically achieved under routine care conditions. To mitigate this, study personnel involved in follow-up were explicitly instructed to avoid acting in a coaching manner during the study, increasing the opportunity to study the online tool and written instructions as stand-alone interventions.

The current study population may represent a healthier and more educated subset of patients. In our cohort, 46–55% of participants reported post-secondary education, compared with approximately 42% in the Swedish general population aged 25–64 years according to national statistics [40]. In addition, a majority of participants reported no comorbidities, which likely reflects both exclusion of patients with severe illness and selection of individuals able to engage in exercise-based rehabilitation and trial procedures.

Although the digital intervention was designed to be user-friendly, factors such as digital literacy and individual motivation were not systematically assessed and may have influenced engagement. Notably, 15 patients were excluded due to lack of access to or proficiency with digital technology, and an additional 10 due to poor understanding of the Swedish language. These exclusions could limit the generalisability of the findings, particularly with respect to populations who may face barriers to digital participation. While the study population was representative of individuals likely to use digital health tools, broader implementation may require alternative delivery formats or support mechanisms to ensure equitable access to vestibular rehabilitation.

### Implications for practice and future research

This study supports the use of vestibular rehabilitation for all patients with AVS, in line with prior evidence and guideline recommendations [13]. The equivalence of outcomes between online and written delivery formats suggests that implementation strategies can prioritise flexibility, accessibility, and patient preference. Given the low uptake of VR in clinical practice, reported to be under 5% among eligible primary care patients [11,12], increasing availability of digital tools may help bridge the gap between evidence and practice, particularly in settings where physiotherapy or specialist follow-up is limited.

Future research should examine whether tailored interventions based on individual risk profiles, such as comorbidities, could enhance treatment effects. Longer follow-up may help determine whether early use of VR reduces the risk of chronic dizziness. Additionally, evaluating guided digital interventions or blended models in the acute setting may further clarify whether added support improves adherence and outcomes.

## Conclusion

In this study, internet-based vestibular rehabilitation was not superior to standardised written instructions in reducing vestibular symptoms after acute onset vertigo. Both interventions led to meaningful symptom improvement, with high compliance and no adverse events related to the exercises. These findings suggest that the choice of delivery format can be guided by patient preference and context, reinforcing the importance of ensuring access to vestibular rehabilitation in all patients after acute vertigo.

## Supporting information

S1 File Consort checklist. This is a point-by-point checklist with manuscript side references for the content in the CONsolidated Standards Of Reporting Trials (CONSORT) checklist.
(PDF)

S2 File LMM Analyses and secondary outcomes. This document reports the Linear mixed models (LMM) analyses of secondary outcomes over time. Secondary outcomes are: Dizziness Handicap Inventory – DHI, including subscales; Balance test performance; subscales of Vertigo Symptom Scale-Short From – VSS-SF; and Timed 25-foot walk test.
(PDF)

S3 Fig Subgroup analyses. This Fig displays a forest plot were interactions between the treatment arm and relevant subgroups are reported.
(PDF)

S4 File Study protocol. The approved Clinical Investigation Plan (CIP) v. 1.6.
(PDF)

S5 File PLOS Human Participants Research Checklist 2025. This is the completed PLOS Human Participants Research Checklist.
(PDF)

S6 File Vestibular rehabilitation excercises. These are the written instruction excercises used in the study. Published with the consent of the depicted actor.
(PDF)

## Abbreviations

ANCOVA: analysis of covariance
AVS: acute vestibular syndrome
BMI: body mass index
BPPV: benign paroxysmal positional vertigo
CI: confidence interval
DHI: dizziness handicap inventory
ENT: ear-nose-throat
eCRF: electronic case report form
ITT: intention-to-treat
PI: principal investigator
PROM: patient-reported outcome measure
RCT: randomised controlled trial
SD: standard deviation
T25-FW: timed 25-foot walk test
VR: vestibular rehabilitation
VSS-SF: vertigo symptom scale short form
VSS-A: autonomic–anxiety subscale
VSS-V: vertigo-balance subscale
vHIT: video head impulse test
