# Supplementary material for: Internet-based vestibular rehabilitation versus written instructions after acute vertigo: A randomised controlled trial
Source: PLoS One. 2026 Jun 12;21(6):e0351092. doi: 10.1371/journal.pone.0351092 (PMC13262863; doi:10.1371/journal.pone.0351092)
Supplement: S2 File — This document reports the Linear mixed models (LMM) analyses of secondary outcomes over time. Secondary outcomes are: Dizziness Handicap Inventory – DHI, including subscales; Balance test performance; subscales of Vertigo Symptom Scale-Short From – VSS-SF; and Timed 25-foot walk test. (PDF) [file pone.0351092.s002.pdf]

S2. Results of linear mixed models (LMM) analyses of secondary outcomes

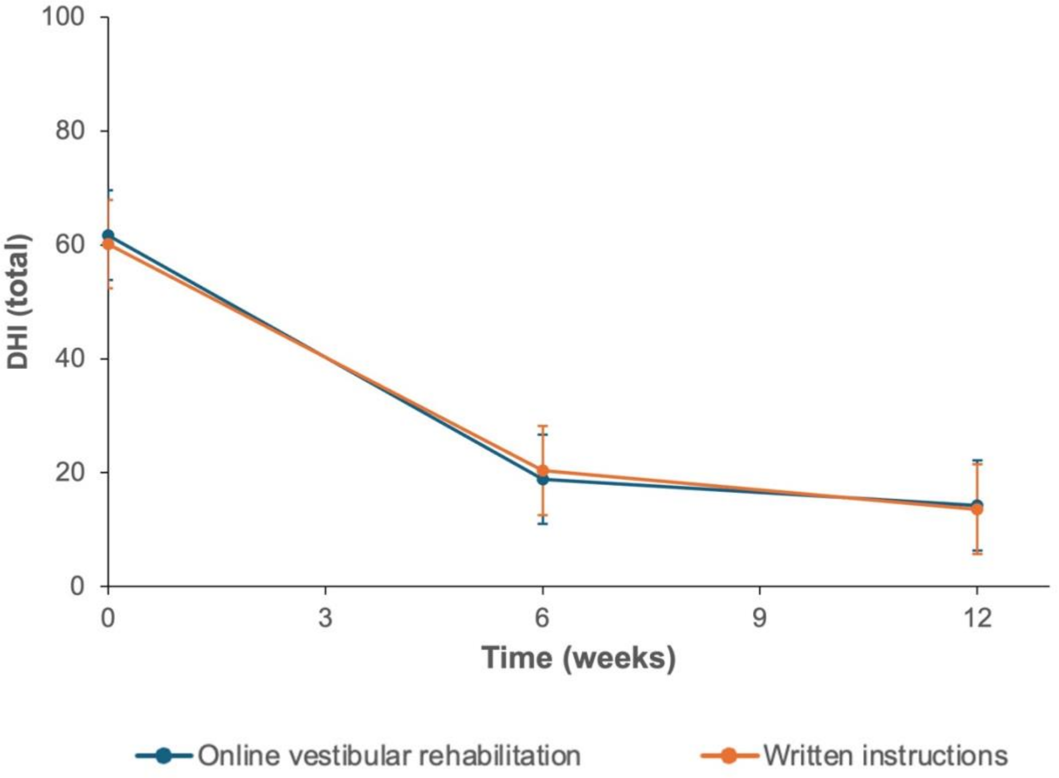

S2.1 Figure. Dizziness Handicap Inventory (DHI) total scores over time by treatment group. Estimated marginal means from linear mixed models for the DHI total score at baseline, 6, and 12 weeks. Analyses were based on the per-protocol cohort. A total of 80 participants in the online vestibular rehabilitation group and 78 in the written instructions group completed the 12-week follow-up. Error bars represent 95% confidence intervals.

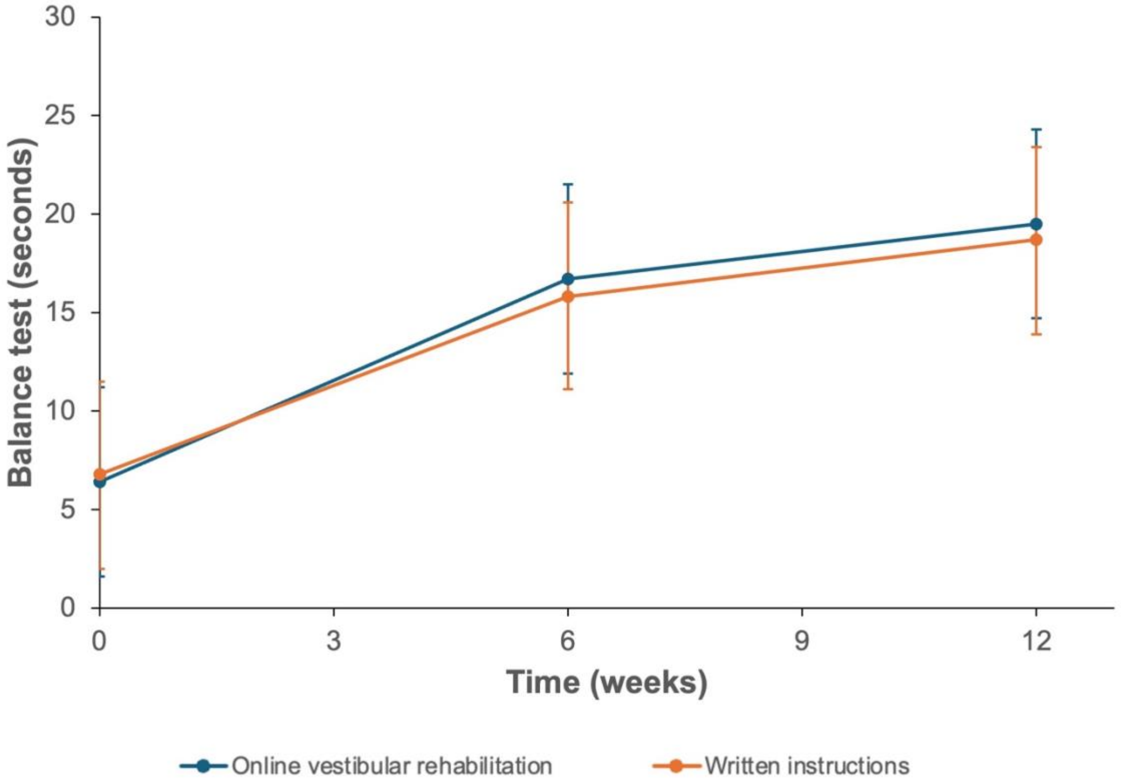

S2.2 Figure. Balance test performance over time by treatment group. Estimated marginal means from linear mixed models for balance duration (in seconds) at baseline, 6, and 12 weeks. Duration was measured as the time participants could maintain balance while standing on a foam pad with eyes closed and performing horizontal head movements at 1 Hz. Analyses were based on the per-protocol cohort. A total of 80 participants in the online vestibular rehabilitation group and 78 in the written instructions group completed the 12-week follow-up. Error bars represent 95% confidence intervals.

S2.1 Table. VSS-SF total and subscale scores over time by treatment group.

	Mean score (SD)		Adjusted mean (95% CI)		Adjusted difference (95% CI)	
	Online VR	Written instructions	Online VR	Written instructions	Online VR vs written instructions	p-value
**VSS-SF**						
Baseline	n=91<br>19.4 (10.3)	n=88<br>20.1 (10.2)	18.2 (14.7–21.8)	19.1 (15.6–22.6)	-0.9 (-3.7–1.9)	0.53
3 weeks	n=89<br>15.7 (9.8)	n=84<br>15.8 (10.9)	14.6 (11.0–18.2)	14.6 (11.1–18.2)	0.0 (-2.8–2.8)	0.99
6 weeks	n=80<br>10.9 (9.3)	n=77<br>12.8 (10.9)	10.1 (6.5–13.7)	11.3 (7.7–14.9)	-1.3 (-4.2–1.6)	0.39
12 weeks	n=81<br>7.5 (9.6)	n=78<br>6.0 (7.0)	6.7 (3.1–10.3)	4.7 (1.1–8.3)	2.1 (-0.8–5.0)	0.16
**VSS-V**						
Baseline	n=92<br>15.1 (8.2)	n=88<br>15.9 (8.5)	14.0 (11.4–16.6)	14.9 (12.3–17.5)	-0.9 (-3.0–1.2)	0.40
3 weeks	n=89<br>11.0 (7.2)	n=84<br>11.4 (8.0)	9.9 (7.3–12.5)	10.3 (7.7–12.9)	-0.4 (-2.5–1.8)	0.46
6 weeks	n=80<br>7.3 (6.7)	n=77<br>8.4 (7.5)	6.3 (3.7–8.9)	7.1 (4.5–9.8)	-0.8 (-3.0–1.4)	0.46
12 weeks	n=81<br>4.3 (6.1)	n=78<br>4.0 (5.1)	3.4 (0.7–6.0)	2.7 (0.1–5.3)	0.6 (-1.6–2.8)	0.56
**VSS-A**						
Baseline	n=93<br>4.1 (3.9)	n=89<br>4.2 (3.3)	4.3 (2.9–5.7)	4.4 (3.0–5.8)	-0.1 (-1.3–1.1)	0.84
3 weeks	n=89<br>4.6 (4.3)	n=84<br>4.4 (4.6)	4.9 (3.5–6.2)	4.5 (3.2–5.9)	0.3 (-0.9–1.5)	0.62
6 weeks	n=80<br>3.7 (3.9)	n=77<br>4.3 (4.9)	3.9 (2.5–5.3)	4.5 (3.1–5.8)	-0.5 (-1.7–0.7)	0.41
12 weeks	n=81<br>3.2 (4.6)	n=79<br>2.0 (2.9)	3.6 (2.1–5.0)	2.2 (0.8–3.6)	1.3 (0.1–2.6)	0.03

Values represent estimated marginal means with 95% confidence intervals from linear mixed models. Models were adjusted for baseline score, age, sex, body mass index, diagnostic group, and study site. A random intercept for site and an AR(1) covariance structure were specified to account for repeated measures.

S2.2 Table. Dizziness Handicap Inventory (DHI) total and subscale scores over time by treatment group.

Secondary outcome measure	Mean score (95% CI) Online VR	Mean score (95% CI) Written instructions	Adjusted mean (95% CI) Online VR	Adjusted mean (95% CI) Written instructions	Adjusted difference (95% CI) Online VR vs written instructions	p−value
**DHI (total)**						
Baseline	n=80<br>62.9 (58.5–67.2)	n=84<br>60.5 (55.5–65.4)	62.8 (54.9–70.6)	61.2 (53.5–68.9)	1.6 (-4.7–7.8)	0.62
6 weeks	n=85<br>19.5 (14.8–24.2)	n=83<br>21.8 (16.9–26.7)	19.4 (11.6–27.2)	21.4 (13.7–29.1)	-2.0 (-8.2–4.3)	0.54
12 weeks	n=80<br>15.0 (9.9–20.0)	n=78<br>15.0 (10.8–19.3)	14.9 (7.0–22.8)	14.8 (7.0–22.6)	0.2 (-6.2–6.5)	0.96
**DHI-P**						
Baseline	n=81<br>19.8 (18.3–21.3)	n=84<br>20.0 (18.4–21.6)	18.8 (16.3–21.2)	19.2 (16.8–21.5)	-0.4 (-2.5–1.7)	0.71
6 weeks	n=85<br>7.2 (5.6–8.8)	n=83<br>8.0 (6.5–9.5)	6.2 (3.8–8.6)	6.9 (4.5–9.3)	-0.7 (-2.8–1.4)	0.52
12 weeks	n=81<br>5.9 (4.2–7.6)	n=79<br>5.5 (4.1–6.9)	4.9 (2.4–7.3)	4.4 (2.0–6.8)	0.5 (-1.7–2.6)	0.67
**DHI-E**						
Baseline	n=85<br>19.0 (17.1–20.9)	n=86<br>17.5 (15.5–19.5)	20.2 (16.9–23.4)	19.0 (15.8–22.2)	1.2 (-1.4–3.7)	0.37
6 weeks	n=86<br>6.3 (4.5–8.1)	n=84<br>7.0 (5.0–9.0)	7.3 (4.0–10.6)	8.0 (4.8–11.3)	-0.7 (-3.3–1.8)	0.58
12 weeks	n=81<br>5.5 (3.4–7.7)	n=79<br>5.2 (3.4–7.0)	6.3 (3.0–9.6)	6.3 (3.0–9.5)	0.0 (-2.6–2.6)	0995
**DHI-F**						
Baseline	n=87<br>23.7 (22.1–25.3)	n=89<br>23.1 (21.3–24.9)	23.6 (20.9–26.4)	23.2 (20.5–25.9)	0.4 (-1.8–2.7)	0.70
6 weeks	n=86<br>6.4 (4.6–8.2)	n=84<br>6.8 (5.0–8.6)	6.4 (3.6–9.2)	6.8 (4.1–9.6)	-0.5 (-2.7–1.8)	0.70
12 weeks	n=80<br>4.2 (2.6–5.8)	n=78<br>4.3 (2.8–5.9)	4.2 (1.4–7.0)	4.4 (1.6–7.1)	-0.2 (-2.5–2.2)	0.88

Values represent estimated marginal means with 95% confidence intervals from linear mixed models adjusted for baseline score, age, sex, BMI, diagnostic group, and site.

S2.3 Table. Balance test performance over time by treatment group.

	Median (IQR), time in seconds		Adjusted mean (95% CI)		Adjusted difference (95% CI)	
Secondary outcome measure	Online VR	Written instructions	Online VR	Written instructions	Online VR vs written instructions	p−value
**Balance test**						
Baseline	n=83<br>2.0 (0.0–6.0)	n=82<br>2.4 (0.1–8.0)	6.4 (1.6–11.2)	6.7 (2.0–11.5)	-0.36 (-3.5–2.79)	0.82
6 weeks	n=86<br>12.0 (2.7–30.0)	n=82<br>11.5 (3.9–30.0)	16.7 (11.9–21.5)	15.8 (11.0–20.6)	0.84 (-2.3–3.98)	0.60
12 weeks	n=80<br>22.0 (6.3–30.0)	n=78<br>20.5 (4.0–30.0)	19.5 (14.7–24.3)	18.6 (13.8–23.4)	0.86 (-2.3–4.07)	0.60

Values represent estimated marginal means with 95% confidence intervals from linear mixed models, adjusted for baseline score, age, sex, BMI, diagnostic group, and site. Duration (in seconds) was measured as the time participants could maintain balance while standing on a foam pad with eyes closed and performing horizontal head movements at 1 Hz.

S2.4 Table. Timed 25-foot walk test results by treatment group.

Secondary outcome measure	Median (IQR), time in seconds		Adjusted geometric mean ratio (95% CI)	
	**Online VR**	**Written instructions**		**p−value**
**T25-FW**				
Baseline	n=89<br>8.6 (6.6–13.8)	n=86<br>7.8 (6.3–10.8)	1.11 (1.00–1.24)	0.06
6 weeks	n=86<br>6.3 (5.1–7.4)	n=83<br>5.6 (4.8–7.1)	1.08 (0.96–1.21)	0.18
12 weeks	n=81<br>6.4 (5.2–7.4)	n=78<br>5.7 (4.5–6.8)	1.09 (0.97–1.22)	0.15

Median time (in seconds) to complete two timed attempts of a 25-foot walk. Values are presented as medians with interquartile ranges. Between-group comparisons were performed on the log scale and are presented as the ratios of the estimated marginal geometric mean (95% CI).
