# Supplementary material for: Internet-based vestibular rehabilitation versus written instructions after acute vertigo: A randomised controlled trial
Source: PLoS One. 2026 Jun 12;21(6):e0351092. doi: 10.1371/journal.pone.0351092 (PMC13262863; doi:10.1371/journal.pone.0351092)
Supplement: S3 Fig — This Fig displays a forest plot were interactions between the treatment arm and relevant subgroups are reported. (PDF) [file pone.0351092.s003.pdf]

S3. Subgroup analyses.

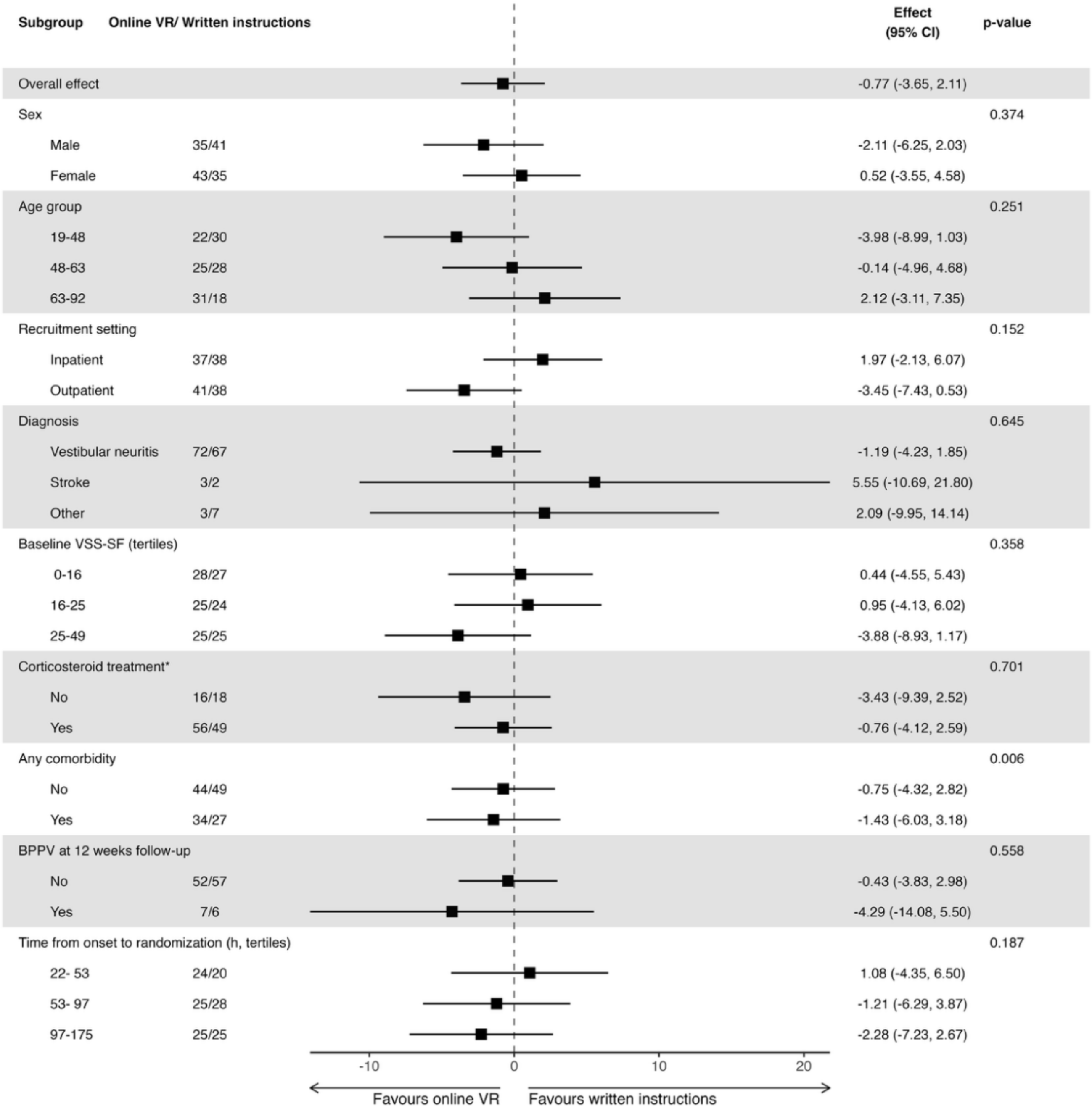

S3 Figure. Forest plot of subgroup analyses. Subgroup analyses showing the treatment effect (difference in change in VSS-SF total score from baseline to 6 weeks) comparing online vestibular rehabilitation (VR) to written instructions. Negative values indicate greater improvement in the online VR group. Effect estimates represent adjusted mean differences with 95% confidence intervals.
