# Supplementary material for: Internet-based vestibular rehabilitation versus written instructions after acute vertigo: A randomised controlled trial
Source: PLoS One. 2026 Jun 12;21(6):e0351092. doi: 10.1371/journal.pone.0351092 (PMC13262863; doi:10.1371/journal.pone.0351092)
Supplement: S4 File — The approved Clinical Investigation Plan (CIP) v. 1.6. (PDF) [file pone.0351092.s004.pdf]

CLINICAL INVESTIGATION PLAN

Internet-based vestibular rehabilitation versus standard care after acute onset vertigo

Web-based Rehab after Acute Vertigo

Version number:	1.6
Date:	2023-04-27

Sponsor and Coordinating Investigator:	*Jonatan Salzer, M.D., Ph.D., Department of clinical sciences, Neurosciences, Umeå University, SE-90187, Umeå, Sweden, +46 70 267 89 64*

Table Of Contents

Signatures 5
Contact information 7
Funding and research agreement 9
List of used acronyms and abbreviations 9
1. Synopsis 11
2. Identification and description of the investigational device 14
2.1. Description of the investigational device 14
2.2. Intended purpose 15
2.3. Manufacturer of the investigational device 15
2.4. Model/type 15
2.5. Summary of required training/experience needed 15
3. Background and justification for the design of the clinical investigation 16
3.1. Background 16
3.2. Evaluation of previous clinical data relevant to the clinical investigation 17
3.3. Description of the clinical development stage 17
4. Risks and clinical benefits of the investigational device and clinical investigation 17
4.1. Expected clinical benefits 17
4.2. Anticipated adverse device effects 17
4.3. Risks associated with participation in the clinical investigation 17
4.4. Steps to be taken to control or mitigate risks 18
4.5. Rationale for benefit-risk ratio 18
5. Objectives and hypotheses of the clinical investigation 18
5.1. The purpose of the clinical investigation 18
5.2. Objectives and endpoints 18
5.3. Hypotheses 20
6. Design of the clinical investigation 20
6.1. General information 20
6.2. Measurements to minimize bias 21
6.3. Measurement of variables 21
6.4. End of the clinical investigation 22

6.5. Comparator and supporting treatment 22
6.6. Subjects 23
6.6.1. Inclusion criteria 23
6.6.2. Exclusion criteria 23
6.6.3. Criteria and procedures for subject withdrawal or discontinuation. 23
6.6.4. Unblinding 24
6.7. Procedures 24
6.8. Monitoring plan 26
7. Informed consent process 27
7.1. General process for informed consent 27
8. Adverse events, adverse device effects and device deficiencies 27
8.1. Definitions 27
8.1.1. Adverse Event 27
8.1.2. Adverse Device Effect 28
8.1.3. Serious Adverse Event 28
8.1.4. Serious Adverse Device Effect 28
8.1.5. Unanticipated Serious Adverse Device Effect 28
8.1.6. Device Deficiency 29
8.2. Recording and Reporting 29
8.2.1. Recording 29
8.2.2. Reporting 29
8.2.3. Assessment of Causality 30
8.3. List of foreseeable Adverse events 31
9. Statistical considerations 32
9.1. Analysis population 32
9.2. Descriptive statistics 32
9.3. Analytical procedures 32
9.4. Sample size calculation 34
9.5. Pass/fail criteria 34
9.6. Interim analysis 34
9.7. Multiplicity control 34
9.8. Subgroup analysis 35
9.9. Missing data 35

9.10. Exploratory analysis and sensitivity analysis 35
9.11. Reporting deviations 35
9.12. Handling of imbalance of subjects per site 35
10. Data management 35
10.1. Case Report Form 35
10.2. Data cleaning and database lock 36
10.3. Data protection 36
10.4. Archiving 37
11. Amendments to the CIP 37
12. Deviations from the CIP 37
13. Device traceability and accountability 37
14. Statements of compliance 38
14.1. Compliance to the investigational plan, good clinical practice, and regulations 38
14.2. Ethical review of the clinical investigation 38
14.3. Insurance 38
15. Premature termination of the clinical investigation 38
16. Publication policy 39
17. Bibliography 39

Principal Investigator

I have read this CIP and agree that it includes all essential information to be able to conduct the study. By signing my name below, I agree to conduct the study in compliance with this Clinical investigation plan, the Declaration of Helsinki, ISO14155:2020 (Good Clinical Practice), and the current national and international regulations governing the conduct of this clinical investigation.

I will submit this CIP and all other important study-related information to the staff members who participate in this study, so that they can conduct the study correctly. I am aware of my responsibility to continuously keep the staff members who work with this study informed and trained.

I am aware that quality control of this study will be performed in the form of monitoring, audit, and possibly inspection.

____

Principal Investigator’s signature Date

____

Printed name

Contact information

**Role**	
Sponsor and Coordinating Investigator	Jonatan Salzer, MD, PhD<br>Docent, Överläkare Neurologi, Umeå<br><br>Department of clinical sciences, Neurosciences, Umeå University, SE-90187, Umeå<br><br>jonatan.salzer@umu.se<br><br>+46 70 267 89 64
Principal Investigator	Solmaz Surano, MD, PhD<br>AT-läkare, Postdoktor, Umeå<br><br>solmaz.surano@umu.se<br><br>+46 70-571 25 15
Principal Investigator	Erik Faergemann, MD<br>ST-läkare Neurologi, Sundsvall<br><br>erik.faergemann@rvn.se<br><br>+46 70-513 24 80
Principal Investigator	Maria Bjurman, PT<br>Fysioterapeut, Sollefteå<br><br>maria.bjurman@rvn.se<br><br>+46 70-368 98 64
Principal Investigator	Linn Wedlund, MD<br>Läkare, ÖNH, Östersund<br><br>linn.wedlund@regionjh.se
Principal Investigator	Hugo Davidsson, MD<br>Läkare, Sahlgrenska<br><br>hugo.davidsson@vgregion.se
Principal Investigator	Fredrik Tjernström, MD, PhD<br>Specialistläkare ÖNH, Lund<br><br>fredrik.tjernstrom@med.lu.se

**Role**	
Principal Investigator	Ellen Lindell, MD, PhD<br>Specialistläkare ÖNH, Borås<br><br>ellen.lindell@gmail.com<br><br>+46 70 420 06 28
Principal Investigator	Tatjana Tomanovic, MD, PhD<br>Överläkare ÖNH, Karolinska<br><br>tatjana.tomanovic@sll.se<br>+46 8 517 738 91
Principal Investigator	Torbjörn Ledin, MD, PhD<br>Professor, Överläkare ÖNH, Linköping<br><br>torbjorn.ledin@liu.se<br><br>+46 73 068 84 21
Principal Investigator	Anette Sörlin, MD<br>Överläkare ÖNH, Sunderbyn<br><br>anette.sorlin@norrbotten.se<br><br>+46 72 700 44 50
Principal Investigator	Jan Mathé, MD, PhD<br>Överläkare neurologi, Capio S:t Göran<br><br>jan.mathe@capiostgoran.se
SAE reporting	Jonatan Salzer, MD, PhD<br><br>Department of clinical sciences, Neurosciences, Umeå University, SE-90187, Umeå<br><br>jonatan.salzer@umu.se<br><br>+46 70 267 89 64
Coordinating monitor	Kliniskt Forskningscentrum<br>Region Västerbotten<br><br>klinisktforskningscentrum@regionvasterbotten.se

Funding and research agreement

This is an investigator-driven study which is fully funded by a Swedish Research Council grant, grant number 2020-00301. The sponsor and site PIs agree to fulfil their respective duties within the study as detailed in the Site Agreement. The sponsor has full access to, and retains all rights to, the data generated in the study. The site PIs participate as co-authors in the publication of the primary endpoint (expected highest impact publication) and may also participate in additional publications based on interest and contribution, as agreed. The Sites will also receive reimbursement for included and fully investigated (including month 3 data collection) study participants as detailed in the Site Agreement.

List of used acronyms and abbreviations

**Abbreviation**	**Term/Explanation**
ADE	Adverse Device Effect
AE	Adverse Event
CIP	Clinical Investigational Plan
CRF	Case Report Form
DD	Device Deficiency
DHI	Dizziness Handicap Inventory
GCP	Good Clinical Practice
IB	Investigator's Brochure
ICMJE	International Committee of Medical Journal Editors
IFU	Instructions for Use
ISO	International Organization for Standardization
ITT	Intention-to-treat = including all data from all subjects who have participated in the study
LVFS	Läkemedelsverkets författningssamling (English: Swedish Medical Products Agency's statutes)
PP	Per Protocol analysis = including only data from subjects who have completed the study completely in accordance with the CIP, with no deviations from the CIP

SADE	Serious Adverse Device Effect
SAE	Serious Adverse Event
USADE	Unanticipated Serious Adverse Device Effect
VSS-SF	Vestibular Symptom Scale – Short Form
vHIT	Video Head Impulse Test
VOR	Vestibulo-ocular reflex

1. Synopsis

Background and rationale:	Acute onset vertigo is common and entails much suffering with persisting symptoms at 3 months after onset in up to half of those afflicted. Vestibular rehabilitation to aid recovery is not readily available. The purpose of this study is to investigate the effects on vertigo symptoms of a 6-week online vestibular rehabilitation tool compared with standard care (written instructions leaflet) after acute onset vertigo.
Investigational device:	Online vestibular rehabilitation tool
Number of subjects:	184
Inclusion criteria (all):	• ≥18 years old; *and*<br>• The subject has given written consent to participate in the study; *and*<br>• New acute onset dizziness or vertigo since ≥24 hours with pathological spontaneous or gaze-evoked nystagmus (i.e., an acute vestibular syndrome, AVS). The nystagmus as described above must be present at investigation between 24 hours and 7 days from onset, spontaneously, gaze-evoked or head-shake evoked and documented; *and*<br>• Screening and inclusion within 7 days of onset of continuous symptoms; *and*<br>• Symptomatic at inclusion
Exclusion criteria (any):	• Pre-existing vestibular disease or neurological disease anticipated to affect the ability to participate in the study or the effect of the intervention. N.B: Recurring AVS with no set diagnosis before inclusion is accepted, as is past transient neurological diseases such as TIA or migraine; *or*<br>• Inability to use the online rehabilitation tool, e.g., due to not having access to a computer, tablet or smartphone, not having access to the internet or lacking in experience with such tools; *or*<br>• Mental inability, reluctance or language difficulties that result in difficulty understanding the meaning of study participation; *or*

	• Medical and/or physical contraindications to making the required head movements (e.g., vertebral dissection) or otherwise participating in the training and testing exercises or data collection; *or*<br>• Medication or other substance intake which can affect the ability to participate in the study or the reliability of the measurement methods. These medications include regular use of: Anticonvulsants, antiemetics/motion sickness medications, benzodiazepines, neuroleptics. Transient corticosteroid and/or antiemetic treatment related to the current vertigo is accepted.
Study objectives:	Primary objective:<br>To compare the effect of the online vestibular rehabilitation tool with standard written instructions after acute onset vertigo on vestibular symptoms.<br><br>Secondary objectives:<br><br>1. Compare the impact of the online vestibular rehabilitation tool with standard written instructions after acute onset vertigo on different aspects of everyday living.<br><br>2. Compare how the online vestibular rehabilitation tool influences the walking ability compared with standard written instructions after acute onset vertigo.<br><br>3. Compare how the online vestibular rehabilitation tool influences the lateral canal vestibulo-ocular reflex (VOR) recovery compared with standard written instructions after acute onset vertigo.<br><br>4. Compare the long-term effects of early vs. delayed online vestibular rehabilitation on vestibular symptoms and mobility.<br><br>5. Compare the effects of online vestibular rehabilitation with standard written instructions on vestibular rehabilitation compliance.<br><br>6. Compare the health economic effects of online vestibular rehabilitation with standard written instructions.<br><br>7. Compare the multi-joint kinematic output data from a portable multi-sensor movement analysis system, with the hip kinematic output data received from the mobile phone app.

	8. Translate and validate the VSS-SF scale from English to Swedish.<br><br>9. Investigate the frequency of benign positional paroxysmal vertigo (BPPV) after AVS at different time points and using different evaluation methods; investigate the correlation between BPPV the DHI, VSS-SF, steps and safety endpoints; and investigate the effect of treatment arm allocation on the risk for BPPV.<br><br>Safety objective(s): Compare the effects on the risk of falls/fractures of online vestibular rehabilitation compared with standard written instructions.
Study endpoints:	Primary endpoint:<br>The vertigo symptom scale short form (VSS-SF) score at 6 weeks after vertigo onset.<br><br>Secondary endpoints:<br>1. The between groups mean dizziness handicap inventory (DHI) score at 6 weeks and 3 months after vertigo onset.<br><br>2a. The between groups changes in timed 25-foot walk test (time duration; T25-FW) from baseline to 6 weeks and 3 months; the between group changes in kinematic and temporal parameters during walking (body sway, range of motion, gait phase durations) at 6 weeks and 3 months after vertigo onset.<br><br>2b. The between groups changes in body sway during the Romberg test, from baseline to 6 weeks and 3 months after vertigo onset.<br><br>3. The between groups changes in video head impulse test (vHIT, site-dependent) measured lateral canal VOR gain and saccades from baseline at 6 weeks and 3 months after vertigo onset.<br><br>4. The between groups mean vertigo symptom scale short form (VSS-SF) score at 12 months after vertigo onset; and the between group pedometer-derived number of steps walked since last visit at 6 weeks and 3 months after vertigo onset.<br><br>5. The between groups mean number of weekly training sessions at 6 weeks.

	6. Register-based search for health economic effects on all levels of care (primary, specialized) and society (sick leave).<br><br>7. The difference in kinematic output between measurement systems (i.e., multi-sensor and mobile app) during the tests specified in 2a and 2b.<br><br>8. The reliability and validity of the Swedish VSS-SF translation.<br><br>9. The frequency (percentage) of participants with:<br>BPPV at 3 months after AVS onset;<br>symptoms indicating BPPV 6 weeks after an AVS using a BPPV specific questionnaire;<br>positional nystagmus (non-BPPV) 3 months after an AVS;<br>positional vertigo (non-BPPV) 3 months after an AVS;<br>BPPV in the treatment vs control group; and<br>the DHI/VSS-SF/steps/safety results differences between the BPPV, suspected BPPV and non-BPPV groups.<br><br>Safety endpoint:<br>The proportion of participants who has experienced falls/fractures since study start up until 6 weeks, 3 months and 12 months; and the number of falls/fractures in each study arm at the same time points.
Planned duration of the clinical investigation:	October 2021 –December 2024

2. Identification and description of the investigational device

2.1. Description of the investigational device

The investigational device is a digital tool (software) for guiding patients through a 6-week vertigo rehabilitation. The main feature of the investigational device is that it during a period of six weeks give the patients customized exercises for each week depending on rehabilitation progress. The investigational device also keeps track of the users' progress and reminds the user when it is time to perform the exercises. The exercises are explained by text and short video instructions. The investigational device is a web application and can be run on any platform that have an internet connection.

Each user will be assigned with a personal login for the tool.

2.2. Intended purpose

The online vestibular rehabilitation tool (device) is a software intended for use with common handheld devices and computers/laptops to facilitate training/rehabilitation during chronic vertigo without ICD diagnostic limitations (as validated in previous studies, references 10, 11, 13) and after acute onset vertigo (as validated during this clinical investigation). Any diagnosis with dizziness or vertigo as a presenting symptom, including vestibular neuritis, cerebrovascular disease, Meniere’s disease, inner ear infections or other disturbances, and more, may present according to the inclusion criteria. However, acutely presenting BPPV patients will not be sought to be included in the investigation and thus not in the intended purpose, as these patients will benefit more appropriately from specific repositioning maneuvers. During the clinical investigation persons with acute onset vertigo (Acute Vestibular Syndrome, AVS) as specified in the inclusion criteria will be targeted for inclusion. As the device (in English and Dutch) has been found effective for use among chronic dizziness patients as well the intended purpose after study completion (if the primary endpoint is met) will be all dizziness/vertigo patients in need of vestibular rehabilitation.

2.3. Manufacturer of the investigational device

Name: Jonatan Salzer
Address: Department of clinical sciences, Neurosciences, Umeå University, SE-90187, Umeå, Sweden
Contact, phone number: jonatan.salzer@umu.se, Mobile: +46 70 267 89 64

2.4. Model/type

The “YrselTräning” research version software version 1.0 will be the version used in the clinical investigation.

2.5. Summary of required training/experience needed

The users will install the investigational device on their own personal devices (laptop, computer, tablet or phone) by following a link provided by the study personnel. An internet connection will be required for the installation process. The investigational device runs on all modern browsers with latest version (3), including but not exclusively: *Apple Safari; iOS, Google Chrome; Android*, *Microsoft Edge*, *Mozilla Firefox.*

As the application is a web application the definition of hardware/software requirements such as Android/iOS/Windows/Mac versions or screen size etc is dependent on the device’s ability to use updated versions of the abovementioned web browsers. The investigational device will not be able to control hardware properties such as screen brightness. The investigational device will be made as accessible as possible with Web Content Accessibility Guidelines (WCAG) in mind.

The instructions for use are given orally by study personnel within the informed consent setting as detailed below (Swedish). A written copy of the instructions will also be provided to the subjects.

Onlineverkyget ”YrselTräning” får enbart användas i den kliniska prövningen Balanskontrollstudien (CIV-21-05-036744).
Om du blir lottad till att använda onlineverktyget kommer du att under de 6 första veckorna i studien göra dagliga övningar (15–20 minuter) samt veckovisa utvärderingar. Efter utvärderingarna anpassas övningarna för den följande veckan utifrån dina framsteg. Onlineverktyget innehåller sex olika enkla övningar där man skakar eller nickar på huvudet med öppna ögon som följer med rörelsen, med slutna ögon och med öppna ögon som fixerar föremål. Även kroppsläget (sittande, stående eller gående) varieras för att maximera effekten av träningen.

Den vestibulära rehabiliteringen via onlineverktyget innebär huvudrörelser motsvarande när man skakar på huvudet (huvudrörelsen som signalerar "nej") och huvudrörelser motsvarande när man nickar ("ja") under olika blick-betingelser (öppna ögon som följer med i rörelsen, slutna ögon, öppna ögon som fixerar föremål) samt olika kroppslägen (sittande, stående, gående). Samtliga dessa övningar är ofarliga och väl testade under lång tid. Samma rörelser rekommenderas i de skrivna standardinstruktionerna som används på många center i landet och som även kommer användas i studien. Obehag i form av illamående och yrsel förekommer vid vestibulär rehabilitering (och är en förutsättning för tillfrisknande) men ett deltagande i studien innebär inte ett ökat obehag jämfört med standardbehandling. Du kommer behöva lägga en del tid på ditt studiedeltagande samt ombedjas svara på frågor kring din hälsa vilket kan uppfattas som integritetskränkande.
Om du upplever problem utöver detta kontakta lokalt ansvarig studiepersonal.

3. Background and justification for the design of the clinical investigation

3.1. Background

Dizziness and vertigo are common reasons for contacting health services accounting for around 3% of all emergency department (ED) visits world-wide,[1-5] and 15% of adults report a problem with dizziness in the last 12 months.[6] In a recent analysis of sick leave in Sweden vestibular disorders were the most common reason for audio-vestibular causes of sick leave.[7, 8] Vertigo is associated with an excessive use of diagnostic imaging and emergency care as well as decreased productivity, mainly due to work absenteeism.[9] There is a lack of knowledge considering full economic evaluations (specialized care, primary care and societal costs).
Vestibular rehabilitation is an evidence-based treatment for chronic dizziness but is used in <5% of target patients in primary care.[10, 11] Vestibular rehabilitation in the form of supervised group exercise therapy (38 sessions lasting 1–1.5 hours) was recently shown to be effective after vestibular neuritis, a common cause of acute onset vertigo.[12] Supervised group therapy interventions are not readily available and there is therefore a need for a more easily accessible tool for vestibular rehabilitation.

3.2. Evaluation of previous clinical data relevant to the clinical investigation

An internet-based vestibular rehabilitation tool (in English and Dutch) has been developed and evaluated for chronic dizziness in primary health care,[10, 13] but not for acute onset vertigo despite data suggesting that persisting symptoms 3 months after onset may occur in 30–50% of acute onset vertigo patients.[14] The current study was therefore designed to translate and validate this freely available vestibular rehabilitation tool (https://balance.lifeguidehealth.org/); and subsequently to test the effectiveness of this tool in a hospital-based cohort of acute onset vertigo patients to prevent chronic vertigo, dizziness and balance disturbances.

3.3. Description of the clinical development stage

Pivotal confirmatory clinical investigation.

4. Risks and clinical benefits of the investigational device and clinical investigation

4.1. Expected clinical benefits

Subjects using the online vestibular rehabilitation tool are expected to recover earlier and to a greater extent from their vertigo based on previous data. All subjects will be given access to the online vestibular rehabilitation tool (at study start for the active treatment arm; 3 months into the study for the control arm). This means that if the online tool works better than standard treatment, all study participants will benefit from this. Since up to 50% of people with acute vestibular syndrome without treatment have persistent symptoms after 3 months, it is likely that effective treatment will lead to reduced suffering, lower risk of falls, lower need for sick leave and higher quality of life.

4.2. Anticipated adverse device effects

Mild discomfort may arise from performing the prescribed exercises (see 4.3). Since these exercises are the same ones as those prescribed during standard care (although with higher expected compliance) no additional ADEs are expected apart from those arising from standard care.

4.3. Risks associated with participation in the clinical investigation

Online vestibular rehabilitation involves head movements corresponding to shaking the head (head movement signaling "no") and head movements corresponding to nodding ("yes") under different gaze conditions (open eyes accompanying the movement, closed eyes, open eyes that fixate objects) and different body positions (sitting, standing, walking). These exercises are harmless and well tried for a long time. The same movements are recommended in the written standard instructions that are used at many centers and that also will be used in the study. Discomfort in the form of nausea and dizziness occurs during vestibular rehabilitation (and is a prerequisite for recovery), but we assess that the study

does not involve an increased discomfort compared with standard treatment for the subjects. The main risk associated with malfunction of the online vestibular rehabilitation tool is inadequate rehabilitation.
Subjects will be expected to invest time on study participation and are asked to answer questions about their health, which can be perceived as violating privacy.

4.4. Steps to be taken to control or mitigate risks

To minimize the risk of technical malfunctioning, the online vestibular rehabilitation tool is developed using state of the art methods for programming and validation.
In addition to online vestibular rehabilitation, only the data collection differs from clinical practice, and this is considered necessary and reasonably extensive to maximize the data exchange but still be feasible.

4.5. Rationale for benefit-risk ratio

The small risks of study participation are justified by the potential benefits of the study results and we conclude that it is ethical to perform this study.

5. Objectives and hypotheses of the clinical investigation

5.1. The purpose of the clinical investigation

Acute onset vertigo is common and entails much suffering with persisting symptoms at 3 months after onset in up to half of those afflicted. Vestibular rehabilitation to aid recovery is not readily available. The purpose of this study is to investigate the effects on vertigo symptoms of a 6-weeks online vestibular rehabilitation tool compared with standard care (written instructions leaflet) after acute onset vertigo. The primary outcome variable is the vertigo symptom scale short form (VSS-SF) score at 6 weeks after vertigo onset. Secondary outcomes include effects of the intervention on activities of daily living, mood/anxiety, vestibular function recovery (vestibulo-ocular reflex gain and saccades) and mobility measures. A change in VSS-SF score of 3 or more has been defined as clinically significant.[10, 11] In an earlier study on a Dutch version of the online vestibular rehabilitation tool on primary care chronic dizziness patients the mean VSS-SF score at three months in the intervention group was 8.1 (SD 7.4), and in the control group 11.5 (9.9).

5.2. Objectives and endpoints

Several relevant aspects of subjective vertigo, everyday living, quality of life, mobility and balance as well as objective physiological measurements of balance system recovery are analysed to provide full coverage of potential effects of the online rehabilitation.

Primary objective	Primary endpoint

Compare the effect of online vestibular rehabilitation with standard written instructions after acute onset vertigo on vestibular symptoms (superiority).	The vertigo symptom scale short form (VSS-SF) score at 6 weeks after vertigo onset.
<u>Secondary objectives</u>	<u>Secondary endpoints</u>
1. Compare the impact of the online vestibular rehabilitation tool with standard written instructions after acute onset vertigo on different aspects of everyday living (superiority).<br><br>2. Compare how the online vestibular rehabilitation tool influences the walking ability and standing balance compared with standard written instructions after acute onset vertigo (superiority).<br><br>3. Compare how the online vestibular rehabilitation tool influences the lateral canal vestibulo-ocular reflex (VOR) recovery compared with standard written instructions after acute onset vertigo (superiority).<br><br>4. Compare the long-term effects of early vs. delayed online vestibular rehabilitation on vestibular symptoms and mobility (superiority).<br><br>5. Compare the effects of online vestibular rehabilitation with standard written instructions on vestibular rehabilitation compliance (superiority).<br><br>6. Compare the health economic effects of online vestibular rehabilitation with standard written instructions (superiority).<br><br>7. Compare the kinematic output data from a portable multi-sensor movement analysis system, with the kinematic output data received from the mobile phone app (comparative and partly superiority).	1. The between groups mean dizziness handicap inventory (DHI) score at 6 weeks and 3 months after vertigo onset.<br><br>2. The between groups changes in timed 25-foot walk test (T25-FW) from baseline to 6 weeks and 3 months; the between groups body sway during standing and walking; and the mobility at 6 weeks and 3 months after vertigo onset.<br><br>3. The between groups changes in video head impulse test (vHIT, site-dependent) measured lateral canal VOR gain and saccades from baseline at 6 weeks and 3 months after vertigo onset.<br><br>4. The between groups mean vertigo symptom scale short form (VSS-SF) score at 12 months after vertigo onset; and the between group pedometer-derived number of steps walked since last visit at 6 weeks and 3 months after vertigo onset.<br><br>5. The between groups mean number of weekly training sessions at 6 weeks.<br><br>6. Health economic effects on all levels of care (primary, specialized) and society (sick leave).<br><br>7. The added-value from using a multi-sensor movement analysis system to receive multi-joint kinematic output, in comparison to using a mobile phone placed on the hip to receive center-of-mass kinematic output.

8. Translate and validate the VSS-SF scale from English to Swedish.<br><br>9. Investigate the frequency of benign positional paroxysmal vertigo (BPPV) after AVS at different time point and using different evaluation methods; investigate the correlation between BPPV and the DHI, VSS-SF, steps and safety endpoints; and investigate the effect of treatment arm allocation on the risk for BPPV.	8. The reliability and validity of the Swedish VSS-SF translation.<br><br>9. The frequency (percentage) of participants with:<br>BPPV at 3 months after AVS onset;<br>symptoms indicating BPPV 6 weeks after an AVS using a BPPV specific questionnaire;<br>positional nystagmus (non-BPPV) 3 months after an AVS; positional vertigo (non-BPPV) 3 months after an AVS;<br>BPPV in the treatment vs control group; and the DHI/VSS-SF/steps/safety results differences between the BPPV, suspected BPPV and non-BPPV groups.
<u>Safety objective</u>	<u>Safety endpoint</u>
Compare the effects on the risk of falls and fractures and any other of online vestibular rehabilitation compared with standard written instructions.	The proportion of participants who has experienced falls/fractures since study start up until 6 weeks, 3 months and 12 months; and the number of falls/fractures in each study arm at the same time points.

5.3. Hypotheses

The group randomized to online vestibular rehabilitation will experience a greater and faster balance recovery measured with the VSS-SF compared with standard care.

6. Design of the clinical investigation

6.1. General information

This is a two-armed evaluator-blinded multicenter randomized controlled clinical trial investigating the effects of online vestibular rehabilitation compared with standard written instructions after acute onset vertigo. The standard care study arm will be granted access to the online rehabilitation tool at 3 months after enrollment.

6.2. Measurements to minimize bias

Subjects will be block-randomized (random blocksize of 4, 6 and 8) 1:1 using site-specific randomization lists generated in R. Randomization will be performed by an independent statistician at Registercentrum Norr. As a precaution measure the lists will contain 4 times as many posts as the expected number of participants at each site. The treatment allocation will be open to the participants and to the site study coordinators, however blinded primary endpoint assessments at week 6 by study coordinators from surrounding sites will be performed as detailed below (section 6.7).

To minimize the risk for an increased effect of rehabilitation mediated by the increased motivation stemming from participating in a clinical trial (in both study arms) the study nurses will be instructed to try to avoid acting as “coaches” and instead concentrate on data collection at the contacts with the participants. This way the effect in the intervention arm will be isolated to the vestibular rehabilitation app, and the effect in the control arm will be isolated to mimic current clinical practice.

Sites will differ according to patient base (e.g., ENT outpatient clinics vs stroke wards). This will entail site mediated differences in participant characteristics. Bias stemming from these differences will be mitigated by including a random effect for site in the primary outcome analysis, and by site stratified randomization.

6.3. Measurement of variables

The VSS-SF measures the frequency of 15 vestibular symptoms on a scale from 0 (no symptoms) to 4 (symptoms most days) during the past month. The score range is 0–60 and a difference from baseline of ≥3 points indicate a clinically significant change.[10, 11] The time frame (1 month) for the symptoms will be left as is for the evaluations at week 6 and after 3 months but for the baseline measurement the participants will be asked to report their symptoms during the last 24 hours; and the two response alternatives indicating the highest frequencies (3, quite often [every week] and 4, very often [almost every day]) will be modified to: 3, quite often (almost every hour) and 4, very often (almost all the time). Although this is a deviation from the original version of the VSS-SF this modification is necessary to capture the short-term status of participants soon after vertigo onset. The modified VSS-SF data will be used for the baseline symptom intensity assessments only.

The DHI (range 0–100 points, 0–30 mild, 31–60 moderate and ≥61 severe dizziness) investigates the effects of dizziness on everyday living. The between group mean DHI score will be compared at each time point as detailed in the statistics section. The scale has subcategories (physical, emotional and functional for DHI) and these will be analyzed separately as exploratory analyses although the total scores will be used for the secondary outcomes.

EQ-5D is an generic instrument which consists of five sub-domains (mobility, self-care, usual activities, pain/discomfort and anxiety/depression) with three severity levels (no, moderate and severe problems).[15] For the current study, the Swedish version EQ-5D-3L will be applied, and the Swedish value set based on experienced health states will be applied to

obtain a health utility score in order to calculate quality-adjusted life years (QALYs).[16] The UK MvH value set will be applied for sensitivity analyses.[17]

The T25-FW data will be collected by using the mean (seconds) of two, timed attempts at walking a clearly marked 25-foot course.

Basic body sway data will be collected by using a locally developed application which utilizes cell phone built-in motion tracking. An enhanced gait analysis with a wearable motion tracking system will be performed at Umeå University Hospital for in-depth measurements.[20, 21]

The motion tracking and gait data consist of sagittal and frontal plane angles and angular velocities. The planes are plotted against each other, and large deviations (quartiles or standard deviations) indicate poor balance. An artificial intelligence paradigm to analyze gait recovery will also be explored.[22]

The VOR gain in each subject is expressed as the mean of ≥5 approved impulses for each lateral canal (right, left); on an aggregated level expressed as the median of VOR means and the changes from baseline at 6 weeks and 3 months will be compared between the study arms (affected side).

BPPV will be investigated by asking questions regarding paroxysmal motion-dependent vertigo at 6 weeks and 3 months and performing dix-hallpike and head roll testing at 3 months.

6.4. End of the clinical investigation

The clinical investigation ends when the last subject has completed the last follow-up. The sponsor will notify the Swedish Medical Products Agency within 15 days after the end of the clinical investigation.

6.5. Comparator and supporting treatment.

The comparator (written instructions) is selected based on that the majority of participating sites uses this as a first line treatment for the target population. Furthermore, the alternative which would be physiotherapist led group training sessions, is not available or feasible to provide in a safe and cost-effective manner.

Swedish guidelines suggest a short-term oral corticosteroid treatment for those with vestibular neuritis although data to support positive long-term effects are lacking.[23, 24] All participants with a presumed vestibular neuritis (i.e. those with a uni-directional horizontal spontaneous or gaze-evoked nystagmus, a positive head impulse test to the opposite direction compared with the nystagmus and no other neurological symptoms or findings) will therefore be offered corticosteroid treatment; 50 mg oral prednisolone daily for 5 days, then taper during 5 days.[24]

The comparator chosen in the study is written instructions for vestibular rehabilitation which is the method used by most participating site for the target population. No national guidelines or pre-existing decision support have been found to support how the target population should be handled and treated regarding vestibular rehabilitation.

6.6. Subjects

6.6.1. Inclusion criteria

To be included in the study, subjects must meet the following criteria:

- ≥18 years old; *and*
- The subject has given written consent to participate in the study; *and*
- New acute onset dizziness or vertigo since ≥24 hours with pathological spontaneous or gaze-evoked nystagmus (i.e., an acute vestibular syndrome, AVS). The nystagmus as described above must be present at investigation between 24 hours and 7 days from onset, spontaneously, gaze-evoked or head-shake evoked and documented; *and*
- Screening and inclusion within 7 days of onset of continuous symptoms; *and*
- Symptomatic at inclusion

6.6.2. Exclusion criteria

Subjects must not be included in the study if any of the following criteria are met:

- Pre-existing vestibular disease or neurological disease anticipated to affect the ability to participate in the study or the effect of the intervention. N.B: Recurring AVS with no set diagnosis before inclusion is accepted, as is past transient neurological diseases such as TIA or migraine; *or*
- Inability to use the online rehabilitation tool, e.g., due to not having access to a computer, tablet or smartphone, not having access to the internet or lacking in experience with such tools; *or*
- Mental inability, reluctance or language difficulties that result in difficulty understanding the meaning of study participation; *or*
- Medical and/or physical contraindications to making the required head movements (e.g., vertebral dissection) or otherwise participating in the training and testing exercises or data collection; *or*
- Medication or other substance intake which can affect the ability to participate in the study or the reliability of the measurement methods. These medications include regular use of: Anticonvulsants, antiemetics/motion sickness medications, benzodiazepines, neuroleptics. Transient corticosteroid and/or antiemetic treatment related to the current vertigo is accepted.

6.6.3. Criteria and procedures for subject withdrawal or discontinuation.

If a subject experiences recurrence of an acute vestibular syndrome within 6 weeks from inclusion the subjects' participation in the study is to be discontinued. The reason for discontinuation is documented in the trial termination form in the eCRF system and any further data collection will be stopped.

If inability to continue study participation (follow up, data collection) due to physical, mental or technical issues arises the site PI is to be immediately notified. The trial termination form in the eCRF system is used to document the reason(s) for and date of discontinuation.

If inability to continue study participation (training) due to physical, mental or technical issues arises the participant will be asked to continue to participate for data collection and observation (ITT analysis).

6.6.4. Unblinding

The clinical investigation is only blinded for the evaluators at week 6 and for the statistical analyses. Therefore, no emergency code breaking protocol is needed.

6.7. Procedures

Figure 1. Study outline – Internet-based vestibular rehabilitation versus standard care after acute onset vertigo, a randomized controlled trial

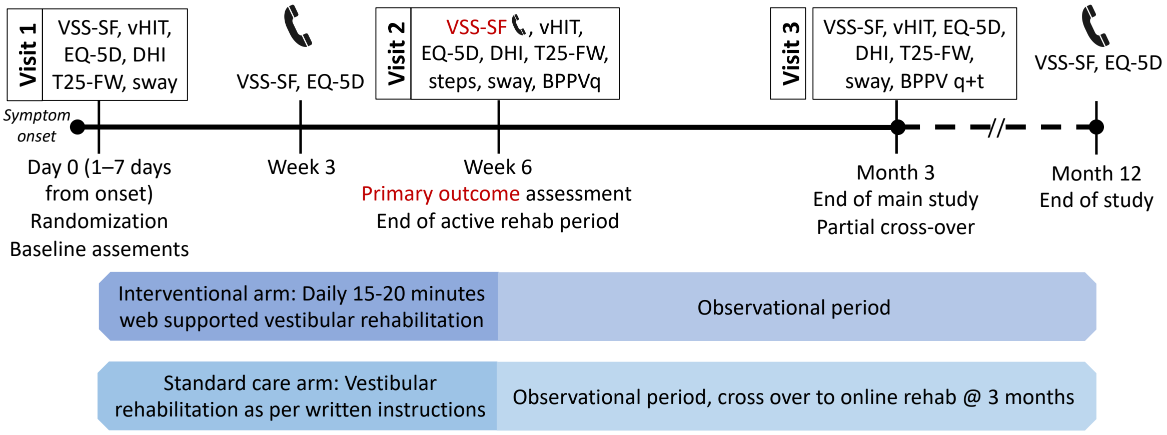

VSS-SF – vertigo symptom scale short form. vHIT – video head impulse test. EQ-5D *– EuroQol five-dimension scale. DHI – dizziness handicap inventory. T25-FW – timed 25-foot walk test. BPPV – benign paroxysmal positional vertigo. q – questions, t – testing.*

Eligible patients will be identified by the site study coordinator using daily emergency department, medical/neurological and ENT wards vigilance (contacting the on-call doctors, contacting the emergency department staff, manual patient list screening including screening of the relevant wards, notice-board ads and information brochures at each department and ward). ENT outpatient clinics identify eligible patients from subacute AVS visits. Local variations of these methods to identify eligible patients will be put in place by each site PI. Eligible patients are screened for participation with a screening checklist (inclusion/exclusion criteria) including screening for nystagmus using videofrenzel goggles (screening of eye movements looking straight forward, 30 degrees to the right, 30 degrees to the left, up, and down); the presence of nystagmus must be documented to be present at least 24 hours after symptom debut.

Subjects will be randomized to the intervention group or control group as detailed in section 6.2.

Intervention: Online 6 weeks vestibular rehabilitation demanding 15–20 minutes of training each day. The intervention aims at maximizing central compensation for the vestibular/balance deficit. It consists of weekly online sessions and daily exercises. The

weekly online sessions consist of educational texts, symptom control techniques, scoring tests and exercise prescriptions for the following week. The exercises for the following week after each online session will be adapted to the user and thus become successively more difficult if the user scores lower discomfort during evaluations, or the opposite if the user scores higher discomfort. There are 6 different exercises in the tool: 1. Head shake with open eyes, gaze following the head; 2. Head nod with open eyes, gaze following the head; 3. Head shake with closed eyes; 4. Head nod with closed eyes; 5. Head shake with open eyes, gaze fixated and; 6. Head nod with open eyes, gaze fixated. These exercises are performed sitting, standing and walking depending on symptom intensity. There are 4 symptom control techniques in the tool in order to deal with secondary anxiety issues related to the vertigo: 1. Controlled breathing; 2. Relaxation; 3. Stress management and; 4. Thought control.[25]

<u>Control</u>: After randomization written instructions with the 6 different exercises from the online rehabilitation tool is handed out and the participant is instructed to increase difficulty if possible. Participants are also given general advice to prevent inactivity and secondary dizziness and fear of motion. Data collection as for the intervention arm. At the end of the core study at 3 months from randomization the standard care study arm participants will also be granted access to the online rehabilitation tool and followed until 12 months.

The Swedish translations of VSS-SF,[18] DHI,[19] and EQ-5D-3L are collected locally at all sites by the site study coordinator. These questionnaire data will be collected by in-person interview at day 0, week 6 and after 3 months, except for VSS-SF at 6 weeks (primary endpoint) which will collected by phone call interview by a blinded study coordinator at either the same site or a different site. In the case of the first 100 included subjects, the VSS-SF at 6 weeks (primary endpoint) will be collected by phone call interview twice, 48 hours apart, as part of the statistical validation of the Swedish translation of the VSS-SF questionnaire [18]. Also, at week 3 and after 12 months VSS-SF and EQ-5D data will be collected by phone call interview (figure 1).

The T25-FW data will be collected by letting the subject walk a clearly marked 25-foot course during follow-up appointments at each site.
Basic body sway data will be collected by each site study coordinator using a locally developed application which utilizes cell phone built-in motion tracking. An enhanced gait analysis with a wearable motion tracking system will be performed at Umeå University Hospital for in-depth measurements.[20, 21]

The participants will be instructed to keep a provided pedometer on them as often as possible during the first 3 months. The total number of steps for the first 6 weeks, and from week 6 to 3 months will be recorded into REDCap will be compared between the study arms at week 6 and after 3 months.

At sites with access to vHIT we will also measure VOR gain and catch-up saccades by performing vHIT at day 0, 6 weeks and 3 months.

The catch-up saccades (yes/no, proportion of impulses with saccades) will also be compared between the study arms at 6 weeks and 3 months (affected side).

The 12 months data collection to capture potential long-term effects of early intervention will be performed by the Umeå University Hospital study coordinator by contacting all participants by telephone at 12 months after inclusion. During this phone call one last round of VSS-SF questionnaire data will be collected (Figure 1).

Table 1. Schedule of events

Visit/contact	Baseline	3 weeks (by phone)	6 weeks	3 months	12 months (by phone)	Comments
Target day	0	21 +/-3	42 +/-3	90 +/-7	360 +/-14	
Eligibility screening, history	x					
Randomization	x					
VSS-SF	x	x	x	x	x	Primary endpoint @6 weeks
EQ-5D	x		x	x	x	
DHI	x		x	x		
T25-FW	x		x	x		
Body sway	x		x	x		
Enhanced body sway analyses	x		x	x		At selected sites only
vHIT	x		x	x		At selected sites only
Steps, falls and fractures			x	x		Total steps, since enrollment or last visit
BPPV questions			x	x		
BPPV testing				x		
Compliance		x	x			Questionnaire data, number of weekly sessions
Trial termination form (local)				x		Last site visit @ 3 months
Trial termination form (central)					x	
Estimated time requirement	150 min	15 min	120 min	120 min	15 min	Total 7 hours per participant

Abbreviations: VSS-SF = vertigo symptom scale short form; EQ-5D = EuroQol five-dimension scale; DHI = dizziness handicap inventory; T25-FW = timed 25-foot walk test; SD = standard deviation; vHIT = video head impulse test.

6.8. Monitoring plan

The study will be monitored by an independent monitor before the study begins, during the study conduct, and after the study has been completed, to ensure that the study is carried out according to the CIP and that data is collected, documented, and reported according to ISO 14155:2020 and applicable ethical and regulatory requirements. Monitoring is performed as per the study's monitoring plan and is intended to ensure that the subject's rights, safety, and well-being are met as well as data in the eCRF are complete, correct, and consistent with the source data.

Monitoring will be performed by visits on site or by telephone call or video contact visits using a secure platform with encrypted communication.
Details of the monitoring activities are outlined in a separate risk-based monitoring plan.

7. Informed consent process

7.1. General process for informed consent

Designated personnel at care units (emergency departments, medical/neurological departments, ear-nose-throat departments/clinics and health centers) briefly inform potential subjects about the study and ask if they are interested and approve of receiving a call to book a visit for more information and written information about the study.

The local study coordinator calls/meets those who are interested and inform about the study and books an inclusion visit.

The principal investigator or delegated members of the study team at each site shall ensure that the subject is given full and adequate oral and written information about the study, its purpose, any risks and benefits as well as inclusion and exclusion criteria. Subjects must also be informed that they are free to discontinue their participation in the study at any time without having to provide a reason. Subjects should be given the opportunity to ask questions and be allowed time to consider the provided information and participation in the clinical investigation. If the person chooses to participate, both the subject and the member of the study team shall sign the informed consent form. A copy of the subject information as well as a copy of the informed consent form shall be provided to the subject. The subject’s signed and dated informed consent must be obtained before performing any study-specific activity in the study. The process shall be documented in the subject’s source documents and the signed informed consents shall be maintained with the essential documents. If new information becomes available that can significantly affect a subject's future health and medical care, that information shall be provided to the affected subject(s) in written form. If new information is added to the study, the subject has the right to reconsider whether he/she will continue their participation.

8. Adverse events, adverse device effects and device deficiencies

8.1. Definitions

8.1.1. Adverse Event

An Adverse Event (AE) is untoward medical occurrence, unintended disease or injury or any untoward clinical signs, including an abnormal laboratory finding, in subjects, users or other persons, in the context of a clinical investigation, whether or not related to the investigational device.

This definition includes events that are anticipated as well as unanticipated events.

This definition includes events occurring in the context of a clinical investigation related to the investigational device, the comparator or the procedures involved.

8.1.2. Adverse Device Effect

An Adverse Device Effect (ADE) is any AE related to the use of an investigational medical device.

This definition includes adverse events resulting from insufficient or inadequate instructions for use, deployment, installation, or operation, or any malfunction of the investigational medical device.

This definition includes any event resulting from use error or from intentional misuse of the investigational medical device.

In the context of the current study the rehabilitation tool is considered equivalent with "Device" above.

8.1.3. Serious Adverse Event

A Serious Adverse Event (SAE) is any AE that led to any of the following:
a) death,
b) serious deterioration in the health of the subject, that resulted in any of the following:
- i. life-threatening illness or injury,
- ii. permanent impairment of a body structure or a body function,
- iii. hospitalization or prolongation of patient hospitalization,
- iv. medical or surgical intervention to prevent life-threatening illness or injury or permanent impairment to a body structure or a body function,
- v. chronic disease,

c) fetal distress, fetal death or a congenital physical or mental impairment or birth defect

8.1.4. Serious Adverse Device Effect

A Serious Adverse Device Effect (SADE) is an ADE that has resulted in any of the consequences characteristic of a serious adverse event.

SAEs related to procedures imposed by the clinical investigation plan but not with the use of the device should not be considered Serious Adverse Device Effects.

In the context of the current study the rehabilitation tool is considered equivalent with "Device" above.

8.1.5. Unanticipated Serious Adverse Device Effect

An Unanticipated SADE is an effect which by its nature, incidence, severity or outcome has not been identified in the current risk assessment. Procedures associated with the use of a device should be addressed in the risk assessment, which makes it possible to determine whether the procedure related SAEs are Unanticipated Serious Adverse Device Effect or not.

SAEs related to procedures imposed by the clinical investigation plan but not with the use of the device should not be considered Serious Adverse Device Effects.

For the anticipated adverse device effects, se section 4.2 above.

In the context of the current study the rehabilitation tool is considered equivalent with "Device" above.

8.1.6. Device Deficiency

A Device Deficiency (DD) is any inadequacy in the identity, quality, durability, reliability, safety or performance of an investigational device, including malfunction, use errors or inadequacy in information supplied by the manufacturer.

In the context of the current study the rehabilitation tool is considered equivalent with "Device" above.

8.2. Recording and Reporting

8.2.1. Recording

The PI or an authorized designee will record in the eCRF:

- all AEs except the events below specified not to be recorded:
    - Vertigo, nausea or mild discomfort during the vestibular rehabilitation, regardless of randomization allocation. These symptoms are common after acute vertigo and during vestibular rehabilitation and all subjects are expected to develop such symptoms.
    - Falls and fractures are recorded and reported as stand-alone endpoints and therefore not included in the AE reporting.
    - VSS-SF (Vertigo Symptom Scale - Short Form) measures the frequency of 15 known vestibular symptoms and these are recorded and reported as endpoints and therefore not included in the AE reporting.
- all SAEs
- all DDs
- any new finding in relation to any of the above-mentioned events.

8.2.2. Reporting

The investigators will report to the sponsor, immediately but not later than 3 calendar days after investigation site study personnel's awareness of the event. The investigators will use the eCRF AE/SAE form for reporting events to the Sponsor.

The sponsor will report, to the Swedish Medical Products Agency (Läkemedelsverket), all of the following:

- any SAE that has a causal relationship with the investigational device or the investigation procedure, or where such causal relationship is reasonably possible.

- any DD that might have led to a SAE if appropriate action had not been taken, intervention had not occurred, or circumstances had been less fortunate; and
- any new findings in relation to any event referred to above.

Reporting by the sponsor will be done by filling out the “Summary Reporting Form” (MDCG 2020-10/1). The form will be filled in/updated for each reportable event or for new findings/updates to already reported events. For events that indicate an imminent risk of death, serious injury, or serious illness and that requires prompt remedial action for other patients/subjects, users or other persons or a new finding to it will be reported immediately, but not later than 2 calendar days after awareness by sponsor of a new reportable event or of new information in relation with an already reported event. Any other reportable events or a new finding/update to it will be reported immediately, but not later than 7 calendar days following the date of awareness by the sponsor of the new reportable event or of new information in relation with an already reported event.

8.2.3. Assessment of Causality

The relationship between each adverse event and the investigational device, the comparator and the investigation procedure will be assessed and recorded by the investigator and sponsor. For assessment of causality, the IB and the risk analysis report will be consulted. The sponsor and investigator will distinguish between SAEs related to the investigational device and those related to the procedures, relatedness to both is possible.

Each SAE will be classified according to four different levels of causality:

1. Not related
   Relationship to the device or procedures can be excluded when:
   - the event has no temporal relationship with the use of the investigational device, or the procedures related to application of the investigational device;
   - the SAE does not follow a known response pattern to the medical device (if the response pattern is previously known) and is biologically implausible;
   - the discontinuation of medical device application or the reduction of the level of activation/exposure - when clinically feasible - and reintroduction of its use (or increase of the level of activation/exposure), do not impact on the serious adverse event;
   - the event involves a body-site or an organ that cannot be affected by the device or procedure;
   - the SAE can be attributed to another cause (e.g., an underlying or concurrent illness/ clinical condition, an effect of another device, drug, treatment or other risk factors);
   - the event does not depend on a false result given by the investigational device used for diagnosis, when applicable;

   In order to establish the non-relatedness, not all the criteria listed above might be met at the same time, depending on the type of device/procedures and the SAE.

2. Possible
   The relationship with the use of the investigational device or the relationship with procedures is weak but cannot be ruled out completely. Alternative causes are also

possible (e.g., an underlying or concurrent illness/ clinical condition or/and an effect of another device, drug or treatment). Cases where relatedness cannot be assessed, or no information has been obtained should also be classified as possible.

3. Probable
The relationship with the use of the investigational device or the relationship with procedures seems relevant and/or the event cannot be reasonably explained by another cause.

4. Causal relationship
The SAE is associated with the investigational device or with procedures beyond reasonable doubt when:
- the event is a known side effect of the product category the device belongs to or of similar devices and procedures;
- the event has a temporal relationship with investigational device use/application or procedures;
- the event involves a body-site or organ that
    o the investigational device or procedures are applied to;
    o the investigational device or procedures have an effect on;
- the serious adverse event follows a known response pattern to the medical device (if the response pattern is previously known);
- the discontinuation of medical device application (or reduction of the level of activation/exposure) and reintroduction of its use (or increase of the level of activation/exposure), impact on the SAE (when clinically feasible);
- other possible causes (e.g., an underlying or concurrent illness/ clinical condition or/and an effect of another device, drug or treatment) have been adequately ruled out;
- harm to the subject is due to error in use

In order to establish the relatedness, not all the criteria listed above might be met at the same time, depending on the type of device/procedures and the SAE.

8.3. List of foreseeable Adverse events

Vertigo, nausea or mild discomfort during the vestibular rehabilitation, regardless of randomization allocation. These symptoms are common after acute vertigo and during vestibular rehabilitation and all subjects are expected to develop such symptoms. Weekly evaluations of these symptoms are included in the online tool and the training intensity is automatically tailored based on symptom intensity. For those randomized to standard care, instructions on lowering training intensity in case of severe discomfort will be given together with the training instructions. The test circumstances at the sites will be designed to provide a safe environment ensuring that no participant will lose balance and fall during walking test.

8.4. Follow-up of adverse events

Serious adverse events and adverse events with a causal relationship to the device that are ongoing at study end will be followed at the site where the participant is registered until the adverse event is either resolved, stable, or persistent. In case of unacceptable adverse events, necessary measures will be taken.
Adverse events and serious adverse events without causal relationship to the device will be followed as part of routine care within the health care system. If necessary the site PI is responsible for notifying the corresponding party responsible for follow up.

9. Statistical considerations

9.1. Analysis population

The primary analysis will be performed using the intention to treat cohort on both primary and secondary outcomes. Per protocol analyses will also be performed as part of the sensitivity analysis.

9.2. Descriptive statistics

Baseline characteristics in both arms will be described using descriptive statistics. The two study arms will be described and compared at baseline using a table including relevant baseline variables. In accordance with the CONSORT protocol no statistical tests of the baseline values will be performed.

9.3. Analytical procedures

Two-sided statistical tests will always be performed. The primary outcome will be analyzed using analysis of covariance (ANCOVA) adjusting for baseline measurements; known predictors of the outcome including diagnostic group, sex, age and baseline symptom intensity in order to gain precision in treatment effect estimates; and inclusion of a random effect for site to control for influence from stratified randomization. The primary endpoint results will be reported as the ANCOVA generated adjusted VSS-SF difference with 95% CI and p-value between the study arms at 6 weeks. The VSS-SF has previously been analyzed in a randomized trial assuming normality.[13] If a clear deviation from normality is detected an ordinal logistic regression ANCOVA will be used for the primary outcome.

For secondary outcomes with repeated measurements we will use linear mixed models analysis for continuous outcome variables and binary logistic mixed effects models for binary outcome variables with random effects for individual and site. These techniques can account for repeated measures within an individual and unbalances from missing data. The intervention effect will be modelled using a time by intervention interaction term.

We will also perform statistical validation of the Swedish translation of the VSS-SF questionnaire, including analyses of reliability (test-retest reliability and internal consistency reliability) and validity; construct validity (convergent and discriminant validity) by investigating item-to-item associations between VSS-SF/VSS-SF domains and DHI; and criterion validity by correlating the domains to DHI and balance tests. For the test-retest

study, the first 100 included subjects will complete the evaluator-blinded 6-week assessments of VSS-SF twice, 48 hours apart, via telephone [18].

Ranked analysis of covariance (ANCOVA) will be used to evaluate intervention effects on T25-FW and VOR gain and saccades, as this method provides robust estimates even though there may be extreme outliers in skewed data. The percentage of participants with sustained improvement in VSS-SF defined as ≥3 points improvement at 6 weeks and 3 months will also be compared using binary logistic regression.

We will report crude and adjusted analyses, adjusting for baseline values of the studied outcome and the prespecified predictors sex, age, level of education, number of chronic diseases and baseline AVS etiology.

Please refer to the schedule of events below for an overview of data collection by time points. Statistician Gabriel Granåsen, Registercentrum Norr, will be responsible for the statistical analysis plan.

Table 2. Aim-specific statistical analysis plan

Analysis	Subjects analyzed	Sample size (15% attrition is expected in power calc.)	Estimated difference based on previous results mean (SD)	Power	Method
Between group mean VSS-SF difference @w3, @w6, @m3, @m12	All participants	92+92	8.1 (7.4) vs 11.5 (9.9)[13]	90%	ANCOVA
Between group mean EQ-5D difference until m3	All participants	92+92	Unknown	Unknown	Mixed models
Between group mean EQ-5D difference @12m	All participants	92+92	Unknown	Unknown	ANCOVA
Between group mean DHI difference until m3	All participants	92+92	24.4 (20.8) vs 29.2 (21.1)[13]	68%	Mixed models
Mean of means T25-FW (seconds) and timed balance tests (seconds) until m3	All participants	92+92	Unknown	Unknown	Mixed models
Between group mean difference of means of vHIT gain (affected side) @w6 and @m3	Participants at sites with access to vHIT	50+50	0.52 (0.24) vs 0.69 (0.25)*[31]	99%	Ranked ANCOVA
Proportion with catch-up saccades	Participants at sites with access to vHIT	50+50	Unknown	Unknown	Binary logistic regression

on vHIT (affected side) @w6 and @m3					
Body sway until m3	All participants	92+92	Unknown	Unknown	Mixed models
Enhanced body sway analysis (gait analysis) until m3	Umeå participants only	25+25	Unknown	Unknown	Mixed models
Total steps since last visit @w6 and @m3.	All participants	92+92	Unknown	Unknown	ANCOVA
Self-reported vestibular rehabilitation compliance until 6w (mean number of training sessions)	All participants	92+92	Unknown	Unknown	Mixed models
Proportion with falls or fractures	All participants	92+92	Unknown	Unknown	Binary logistic regression
Proportion with BPPV	All participants	184	Estimated frequency 10%	N/A	Binary logistic regression

Abbreviations: VSS-SF = vertigo symptom scale short form; EQ-5D = EuroQol five-dimension scale; DHI = dizziness handicap inventory; T25-FW = timed 25-foot walk test; SD = standard deviation; vHIT = video head impulse test; @w6 = at 6 weeks; @m3 = at 3 months; @m12 = at 12 months.

*Power calculations based on mean gain differences before and after vestibular rehabilitation

9.4. Sample size calculation

A change in VSS-SF score of 3 or more has been defined as clinically significant.[10, 11] In an earlier study on a Dutch version of the online vestibular rehabilitation tool on primary care chronic dizziness patients the mean VSS-SF score at three months in the intervention group was 8.1 (SD 7.4), and in the control group 11.5 (SD 9.9). In order to detect a difference in VSS-SF score of 3, assuming a standard deviation of 9 and a correlation between pre and post measurements of 0.6 with 90% power, alpha 5%, we would need n=156 participants, 78 in each arm, using the Shieh sample size calculation method implemented into the Superpower package.[33] Assuming a 15% drop out rate the sample size needs to be increased to n=184 to preserve power. Conservative observational data suggest a yearly incidence of 36 patients with acute onset vertigo and nystagmus per 100,000 inhabitants in the Umeå University uptake area.[5, 30]

9.5. Pass/fail criteria

Pass: The study group randomized to the online vestibular rehabilitation scores mean ≥3 VSS-SF points lower compared with the standard care group at 6 weeks.

9.6. Interim analysis

An interim drop-out rate analysis performed on 25th April 2023 after 138 included subjects (of which 129 have reached 6 weeks) supports the initial drop-out rate assumptions.

9.7. Multiplicity control

No adjustment for multiplicity will be performed as the study has only one primary endpoint.

9.8. Subgroup analysis

Subgroup analyses will be performed by assessing for interaction by:

a) baseline diagnostic group (categorical), i.e., unilateral vestibular deficit (most often vestibular neuritis), cerebrovascular vertigo (stroke) and vertigo NOS.

b) sex (categorical)

c) age (continuous)

d) study site (categorical)

e) baseline symptom severity – VSS-SF (continuous)

f) BPPV diagnosis or not at 3 months (categorical)

9.9. Missing data

To preserve the ITT principle for the primary endpoint we will use, hierarchically, (1) the 6 weeks assessment of VSS-SF; if missing, (2) the 3 weeks assessment; and (3) the 3 months assessment. A sensitivity analysis will be performed by comparing the primary analysis results with and without the imputed values from the 3 weeks and 3 months values.

9.10. Exploratory analysis and sensitivity analysis

As a sensitivity analysis to explore robustness of data we will exclude the 10% with the lowest numbers of self-reported weekly training sessions in each study arm.

9.11. Reporting deviations

Any relevant deviations from the original statistical analysis plan as outlined in this document will be reported to the medical products agency.

9.12. Handling of imbalance of subjects per site

Randomization will be site-stratified. The primary effects analysis will include a random effect for site to control for influence from stratified randomization.

10. Data management

Subjects who participate in the study are coded with a specific study identification number. All subjects are registered in a subject identification list (subject enrolment and identification list) that connects the subject’s name and personal number with a study identification number. All data will be registered, managed, and stored in a manner that enables correct reporting, interpretation, and verification.

10.1. Case Report Form

To capture data in the study we will be using an in-house developed eCRF (REDCap, https://projectredcap.org). Data is stored on a secure dedicated server. All study site data

collection will be performed through REDCap apart from site specific screening lists subject identification lists which will be kept, managed, and stored in a manner that enables correct reporting, interpretation, and verification. This also applies to the EQ-5D-3L questionnaires (interview, paper format) which are used for primary data collection before data entry into REDCap. All activity in the eCRF system is logged and time stamped.

10.2. Data cleaning and database lock

Study data will be reviewed for completeness by the data monitoring unit through eCRF review every sixth months and missing data queries sent out to the local PIs. When the last participant has undergone the 12 months follow-up phone call any remaining queries will be resolved as above, followed by database lock according to the REDCap protocol at Umeå University.

10.3. Data protection

If any part of the data is handled by any other organization, inside or outside the EU, appropriate agreements and/or other documentation will be established, to ensure that the data processing is performed in accordance with the provisions of the General Data Protection Regulation (EU ordinance 2016/679, GDPR) and other relevant legislation, before any data transfer takes place.

The content of the informed consent form complies with relevant integrity and data protection legislation. In the subject information and the informed consent form, the subject will be given complete information about how collection, use and publication of their study data will take place. The subject information and the informed consent form will explain how study data are stored to maintain confidentiality in accordance with national data legislation. Data is stored on a secure dedicated server at Umeå University. The server is maintained by the central IT-unit at Umeå University and backed-up regularly.

All study site data collection will be performed through the eCRF REDCap, a system with audit-trail to prevent unauthorized change of data. Access to REDCap is approved by the site PI and granted by the study organization at Umeå University and each log-in requires a two-factor authentication to protect the integrity of subject information. Site-specific screening lists and subject identification lists will be kept and managed in a secure manner, and stored in a locked cabinet, without access for unauthorized persons.

All information processed by the sponsor will be pseudonymized and identified with a study-ID.

The informed consent form will also explain that for verification of the data, authorized representatives of the sponsor, as well as relevant authority, may require access to parts of medical records or study records that are relevant to the study, including the subject’s medical history. Sponsor representatives must be approved by the person responsible for the medical records at the health care institution and sign a secrecy agreement to gain access to the subject´s medical records. The records cannot leave the health care institution premises.

In case of a breach in data protection, the established routines for each concerned organization will be followed to minimize the consequences.

10.4. Archiving

The PIs and sponsor will maintain the essential clinical investigation documents in the investigation site files archive and sponsor files archive, respectively. The sponsor shall keep all documentation and data for at least 10 years after the rehabilitation tool removal from the market. The PI will archive all local study documentation for at least 10 years or as long as stipulated by the local institution.

11. Amendments to the CIP

Amendments to the CIP will be agreed upon between the sponsor and study group. Substantial amendments will be filed to the Swedish Ethical Review Authority (Etikprövningsmyndigheten) and the Swedish Medical Products Agency, if applicable, for approval before implementation.

12. Deviations from the CIP

Investigator(s) are not allowed to deviate from the CIP except if it is for the protection of the subject´s rights, safety, or well-being under emergency circumstances.
All such deviations should be documented and reported to the sponsor as soon as possible.

All deviations shall be documented with an explanation and reported to the sponsor in a note-to-file report integrated in the eCRF. Deviations will be reviewed by the sponsor and reported to the appropriate regulatory bodies as required.

13. Device traceability and accountability

The online rehabilitation tool will only be used in the clinical investigation and according to the clinical investigation plan during the study. The sponsor provides the site with written instructions (study documentation) and technical support.

The investigators subject identification lists will be used to keep record of the users of the online rehabilitation tool. This record will include: Study ID, name, personal identification number, and the date of enrollment.

Each user of the online rehabilitation tool will be provided with a username and access code to gain access to the tool.

After end of study and in case of primary endpoint positive results the tool will be made available through an established easily accessible channel to maximize outreach. The details regarding this will be established during the study and presented together with the final study report submitted to the Swedish Medical Products Agency no later than one year after study conclusion.

14. Statements of compliance

14.1. Compliance to the investigational plan, good clinical practice, and regulations

The clinical investigation will be conducted in accordance with the clinical investigation plan, the ethical principles of the Declaration of Helsinki, the principles of ISO 14155:2020 and current national and international regulations governing this clinical investigation. This is to ensure the safety and integrity of the study subjects as well as the quality of the data collected.

14.2. Ethical review of the clinical investigation

The clinical investigation will not commence until written approval/favourable opinion from the Swedish Ethical Review Authority and the Swedish Medical Products Agency have been received.

The final version of the informed consent form and other information provided to subjects, must be approved or given a written positive opinion by the Swedish Ethical Review Authority and the Swedish Medical Products Agency. The Swedish Ethical Review Authority and the Swedish Medical Products Agency must be informed of any changes in the study CIP in accordance with the current requirements.

14.3. Insurance

The care provided in this study is conducted by the regions and the participants are thereby protected by the Swedish Patient Insurance provided by LÖF.

15. Premature termination of the clinical investigation

The sponsor may suspend or prematurely terminate either the clinical investigation at an individual investigation site or the entire clinical investigation for significant and documented reasons. The Swedish Ethical Review Authority, or the Swedish Medical Products Agency may suspend or prematurely terminate the clinical investigation at the applicable investigation sites.

If suspicion of an unacceptable risk to patients arises during the clinical investigation, or when so instructed by the Swedish Medical Products Agency, the sponsor will suspend the clinical investigation while the risk is assessed. The sponsor will terminate the clinical investigation if an unacceptable risk is confirmed. The sponsor will inform all investigators.

The sponsor shall consider terminating or suspending the participation of a particular investigation site or investigator in the clinical investigation if monitoring or auditing identifies serious or repeated deviations on the part of an investigator. If the suspension or premature termination was in the interest of safety, the sponsor shall inform all other principal investigators.

If, in the opinion of the investigator, the clinical observations in the study suggest that it may be unsafe to continue the study at the site, the investigator may terminate participation in the study after consultation with the sponsor. A written statement fully documenting the reasons for such termination will be provided to the sponsor. If the study is prematurely terminated, the investigators should promptly inform the patients and take necessary steps to finalize their engagement in the study. A final study phone call with an offer of a visit will be made as soon as possible after the decision to terminate the study has been taken. All relevant study material must be collected, and accountability completed.

If the study is interrupted or terminated prematurely the sponsor will report to the Swedish Medical Products Agency within 15 days together with a justification. If the sponsor has temporarily halted or prematurely terminated the study on safety grounds, the Swedish Medical Products Agency will be informed within 24 hours.

16. Publication policy

The clinical investigation will be registered in a publicly accessible database before the start of recruitment activities and the content will be updated throughout the conduct of the clinical investigation and the results entered at completion of the clinical investigation.

A clinical investigation report will be prepared within one year of the end of the clinical investigation or within three months of the early termination or temporary halt, irrespective of the results. An easily understandable summary will also be prepared. The clinical investigation report and summary will be submitted to the Swedish Medical Products Agency.

The sponsor will, together with the local PIs, prepare and submit a scientific report of the clinical investigation results for publication in a scientific journal within two years of the end of the clinical study. The ICMJE authorship criteria will be used and all participating PIs offered to participate in the interpretation of the results and writing of the report regarding the main outcome.

17. Bibliography

1. Kerber KA, Meurer WJ, West BT, et al. Dizziness presentations in U.S. emergency departments, 1995-2004. Acad Emerg Med 2008; 15: 744-750.

2. Newman-Toker DE, Hsieh YH, Camargo CA, Jr., et al. Spectrum of dizziness visits to US emergency departments: cross-sectional analysis from a nationally representative sample. Mayo Clin Proc 2008; 83: 765-775.

3. Kroenke K, Hoffman RM and Einstadter D. How common are various causes of dizziness? A critical review. South Med J 2000; 93: 160-167; quiz 168.

4. Royl G, Ploner CJ and Leithner C. Dizziness in the emergency room: diagnoses and misdiagnoses. Eur Neurol 2011; 66: 256-263.

5. Ljunggren M, Persson J and Salzer J. Dizziness and the Acute Vestibular Syndrome at the Emergency Department: A Population-Based Descriptive Study. Eur Neurol 2018; 79: 5-12.

6. Kerber K, Callaghan B and SA. T. Dizziness Symptom Type Prevalence and Overlap: A US Nationally Representative Survey. American journal of medicine 2017, 130(12):1465 e1-e9.

7. Friberg E, Rosenhall U and Alexanderson K. Sickness absence due to otoaudiological diagnoses; a descriptive nationwide study. BMC Public Health 2013; 13: 635.

8. Friberg E, Jansson C, Mittendorfer-Rutz E, et al. Sickness absence due to otoaudiological diagnoses and risk of disability pension: a nationwide Swedish prospective cohort study. PLoS One 2012; 7: e29966.

9. Kovacs E, Wang X and Grill E. Economic burden of vertigo: a systematic review. Health Econ Rev 2019; 9: 37.

10. Geraghty AWA, Essery R, Kirby S, et al. Internet-Based Vestibular Rehabilitation for Older Adults With Chronic Dizziness: A Randomized Controlled Trial in Primary Care. Ann Fam Med 2017; 15: 209-216.

11. Yardley L, Donovan-Hall M, Smith HE, et al. Effectiveness of primary care-based vestibular rehabilitation for chronic dizziness. Ann Intern Med 2004; 141: 598-605.

12. Tokle G, Morkved S, Brathen G, et al. Efficacy of Vestibular Rehabilitation Following Acute Vestibular Neuritis: A Randomized Controlled Trial. Otol Neurotol 2020; 41: 78-85.

13. van Vugt VA, van der Wouden JC, Essery R, et al. Internet based vestibular rehabilitation with and without physiotherapy support for adults aged 50 and older with a chronic vestibular syndrome in general practice: three armed randomised controlled trial. BMJ 2019; 367: l5922.

14. Heinrichs N, Edler C, Eskens S, et al. Predicting continued dizziness after an acute peripheral vestibular disorder. Psychosom Med 2007; 69: 700-707.

15. Group. E. EQ-5D., https://euroqol.org/ (accessed Oct 3 2020).

16. Burstrom K, Sun S, Gerdtham UG, et al. Swedish experience-based value sets for EQ-5D health states. Qual Life Res 2014; 23: 431-442.

17. Dolan P. Modeling valuations for EuroQol health states. Med Care 1997; 35: 1095-1108.

18. Wilhelmsen K, Strand LI, Nordahl SHG, et al. Psychometric properties of the Vertigo symptom scale - Short form. BMC Ear Nose Throat Disord 2008; 8: 2.

19. Jarlsäter S and Mattsson E. Test of reliability of the Dizziness Handicap Inventory and The Activities-specific Balance Confidence Scale for Use in Sweden. Advances in Physiotherapy 2003; 5: 137-144.

20. Kim SC, Kim JY, Lee HN, et al. A quantitative analysis of gait patterns in vestibular neuritis patients using gyroscope sensor and a continuous walking protocol. J Neuroeng Rehabil 2014; 11: 58.

21. Grip H, Nilsson KG, Hager CK, et al. Does the Femoral Head Size in Hip Arthroplasty Influence Lower Body Movements during Squats, Gait and Stair Walking? A Clinical Pilot Study Based on Wearable Motion Sensors. Sensors (Basel) 2019; 19

22. Ahmadi SA, Vivar G, Frei J, et al. Towards computerized diagnosis of neurological stance disorders: data mining and machine learning of posturography and sway. J Neurol 2019; 266: 108-117.

23. Fishman JM, Burgess C and Waddell A. Corticosteroids for the treatment of idiopathic acute vestibular dysfunction (vestibular neuritis). Cochrane Database Syst Rev 2011: CD008607.

24. Uddman R and Davidsson Å. Internetmedicin, Yrsel (Balansstörning), https://www.internetmedicin.se/page.aspx?id=1039 (2019, accessed June 29th 2019).

25. Cousins S, Kaski D, Cutfield N, et al. Predictors of clinical recovery from vestibular neuritis: a prospective study. Ann Clin Transl Neurol 2017; 4: 340-346.

26. MF D, M S, GW T, et al. Methods for the economic evaluation of health care programs. Oxford University Press, 2005.

27. H G, JA D, SS S, et al. Economic evaluation in clinical trials. Second Edition ed.: Oxford University Press, 2015.

28. Keating C, Neovius M, Sjoholm K, et al. Health-care costs over 15 years after bariatric surgery for patients with different baseline glucose status: results from the Swedish Obese Subjects study. Lancet Diabetes Endocrinol 2015; 3: 855-865.

29. Neovius M, Simard JF, Askling J, et al. How large are the productivity losses in contemporary patients with RA, and how soon in relation to diagnosis do they develop? Ann Rheum Dis 2011; 70: 1010-1015.

30. Sandlund MG, Diamant A, Granasen G, et al. Effectiveness of care in acute dizziness presentations. Eur Arch Otorhinolaryngol 2019; 276: 2389-2396.

31. Navari E, Cerchiai N and Casani AP. Assessment of Vestibulo-ocular Reflex Gain and Catch-up Saccades During Vestibular Rehabilitation. Otol Neurotol 2018; 39: e1111-e1117.

32. Gawrowska 2020. Usefulness of Usefulness of Mobile Devices in the Diagnosis and Rehabilitation of Patients with Dizziness and Balance Disorders: A State of the Art Review. Clinical Interventions in Aging. 2020:15 2397:2406

33. Shieh, Gwowen 2020. Power Analysis and Sample Size Planning in ANCOVA Designs. Psychometrika. 2020; 85: 101–20
