# Supplementary material for: Internet-based vestibular rehabilitation versus written instructions after acute vertigo: A randomised controlled trial
Source: PLoS One. 2026 Jun 12;21(6):e0351092. doi: 10.1371/journal.pone.0351092 (PMC13262863; doi:10.1371/journal.pone.0351092)
Supplement: S6 File — These are the written instruction excercises used in the study. Published with the consent of the depicted actor. (PDF) [file pone.0351092.s006.pdf]

Version 1, 2024-08-27

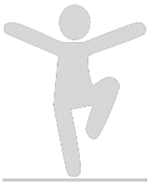

Vestibular rehabilitation exercises

Instructions

The exercises are intended for the treatment of dizziness. They aim to facilitate balance system recovery. Perform the exercises twice a day for 6 weeks. Start seated in a safe and quiet place.

The exercises come in different difficulty levels, A – **EASY**, B – **MEDIUM**, C – **DIFFICULT**. Start with the two easiest exercises (exercises A on page 1 and page 2) and gradually increase the difficulty as you recover. To further increase difficulty, you should try doing the exercises while standing and walking if this can be done safely. However, for safety reasons, you should not perform the exercises with your eyes closed while walking. It is normal to feel dizzy during, and for a short while after, the exercises.

A. Shake **EASY**

1. Shake your head back and forth to the right and to the left 10 times, or as fast as you can, during 10 seconds. Look in the direction of your nose and turn your head as far as it will go without causing discomfort.
2. Rest for 10 seconds and repeat the exercise.

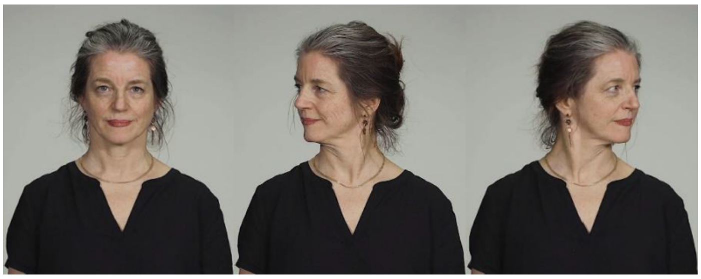

B. Shake – eyes closed **MEDIUM**

1. Close your eyes.
2. Shake your head back and forth to the right and to the left 10 times, or as fast as you can, during 10 seconds. Keep your eyes closed and turn your head as far as you can without causing discomfort.
3. Rest for 10 seconds and repeat the exercise.

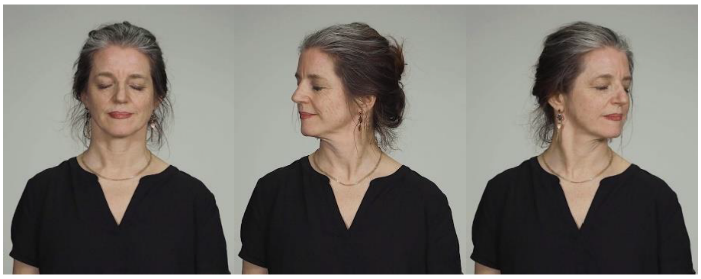

C. Shake – stare **DIFFICULT**

1. Hold one finger in front of you, pointing up.
2. Shake your head back and forth to the right and to the left 10 times or as fast as you can, during 10 seconds. Lock your gaze to your finger and turn your head as far as it will go without causing discomfort.
3. Rest for 10 seconds and repeat the exercise.

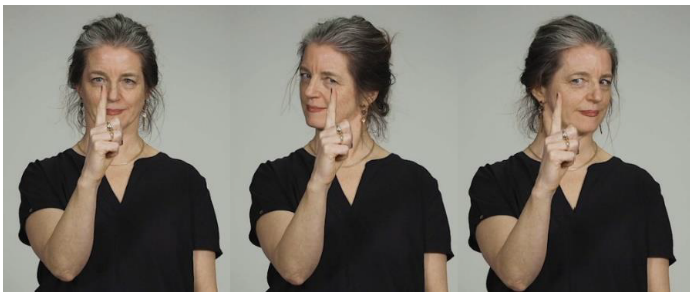

A. Nod **EASY**

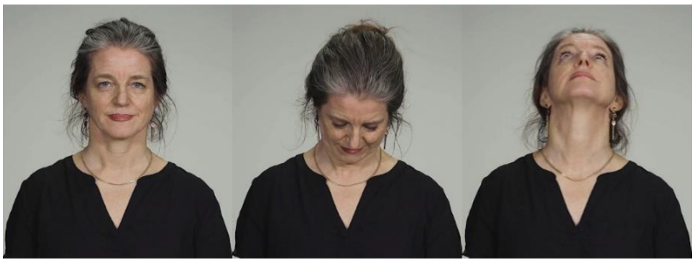

1. Nod your head up and down 10 times, or as fast as you can, during 10 seconds. Look in the direction of your nose and tilt your head as far as it will go without causing discomfort.
2. Rest for 10 seconds and repeat the exercise.

B. Nod – eyes closed **MEDIUM**

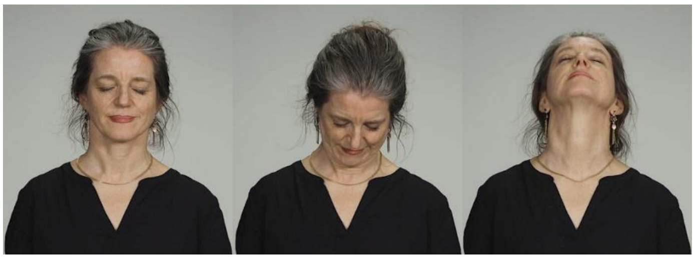

1. Close your eyes.
2. Nod your head up and down 10 times, or as fast as you can, during 10 seconds. Keep your eyes closed and tilt your head as far as it will go without causing discomfort.
3. Rest for 10 seconds and repeat the exercise.

C. Nod – stare **DIFFICULT**

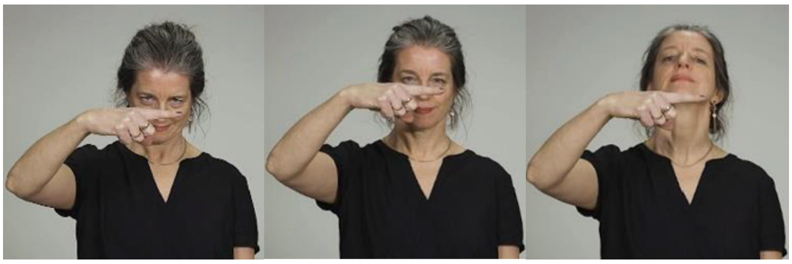

1. Hold one finger in front of you and point straight to the side.
2. Nod your head up and down 10 times, or as fast as you can, during 10 seconds. Lock your gaze to your finger and tilt your head as far as it will go without causing discomfort.
3. Rest for 10 seconds and repeat the exercise.

The exercises have been tested and evaluated in a clinical trial:
Surano S, Grip H, Öhberg F, Karlsson M, Faergemann E, Bjurman M, Davidsson H, Ledin T, Lindell E, Mathé J, Tjernström F, Tomanovic T, Granåsen G, Salzer J. *Trials*. 2022;23:496.

NOTE: Consult your doctor before performing the exercises. These instructions are not a substitute for the advice given to you by your healthcare provider. Contact your healthcare provider if you become acutely ill, develop new symptoms or have questions regarding your illness. Region Västerbotten and Umeå University take no responsibility for any discomfort or complications that may arise during vestibular rehabilitation.
