# Supplementary material for: Internet-based vestibular rehabilitation versus written instructions after acute vertigo: A randomised controlled trial
Source: PLoS One. 2026 Jun 12;21(6):e0351092. doi: 10.1371/journal.pone.0351092 (PMC13262863; doi:10.1371/journal.pone.0351092)
Supplement: Peer review history

# Author comment: Author response to Decision Letter 0

**DOI:** 10.1371/journal.pone.0351092.r001

**Transfer Alert**

This paper was transferred from another journal. As a result, its full editorial history (including decision letters, peer reviews and author responses) may not be present.

19 Mar 2026

# Peer review: Decision Letter 0

**DOI:** 10.1371/journal.pone.0351092.r002

**Authors:** Jeyasakthy Saniasiaya

21 May 2026

Internet-based vestibular rehabilitation versus written instructions after acute vertigo: a randomised controlled trial

PONE-D-26-13474

Dear Dr. Salzer,

We’re pleased to inform you that your manuscript has been judged scientifically suitable for publication and will be formally accepted for publication once it meets all outstanding technical requirements.

Within one week, you’ll receive an e-mail detailing the required amendments. When these have been addressed, you’ll receive a formal acceptance letter and your manuscript will be scheduled for publication.

An invoice will be generated when your article is formally accepted. Please note, if your institution has a publishing partnership with PLOS and your article meets the relevant criteria, all or part of your publication costs will be covered. Please make sure your user information is up-to-date by logging into Editorial Manager at Editorial Manager® and clicking the ‘Update My Information' link at the top of the page. For questions related to billing, please contact billing support.

If your institution or institutions have a press office, please notify them about your upcoming paper to help maximize its impact. If they’ll be preparing press materials, please inform our press team as soon as possible -- no later than 48 hours after receiving the formal acceptance. Your manuscript will remain under strict press embargo until 2 pm Eastern Time on the date of publication. For more information, please contact onepress@plos.org.

Kind regards,

Jeyasakthy Saniasiaya, MD, MMed ORLHNS, FEBORLHNS

Academic Editor

PLOS One

Journal requirements:

When submitting your revision, we need you to address these additional requirements.

1. Please ensure that your manuscript meets PLOS ONE's style requirements, including those for file naming. The PLOS ONE style templates can be found at

https://journals.plos.org/plosone/s/file?id=wjVg/PLOSOne_formatting_sample_main_body.pdf and

https://journals.plos.org/plosone/s/file?id=ba62/PLOSOne_formatting_sample_title_authors_affiliations.pdf

2. Thank you for stating the following financial disclosure:

“This research was funded by the Swedish Research Council (Vetenskapsrådet; https://www.vr.se), grant number 2020-00301, awarded to Jonatan Salzer (J.S.), and through a regional agreement between Umeå University and Region Västerbotten (ALF; https://alfnorra.se/om-alf/regionalt-alf-avtal/). The funding supported the conduct of the research project but was awarded independently of the authors' institutions. Open access funding is provided by Umeå University.”

Please state what role the funders took in the study.  If the funders had no role, please state: "The funders had no role in study design, data collection and analysis, decision to publish, or preparation of the manuscript."

If this statement is not correct you must amend it as needed.

Please respond by return e-mail so that we can amend your financial disclosure and competing interests on your behalf.

3. In the online submission form you indicate that your data is not available for proprietary reasons and have provided a contact point for accessing this data. Please note that your current contact point is a co-author on this manuscript. According to our Data Policy, the contact point must not be an author on the manuscript and must be an institutional contact, ideally not an individual. Please revise your data statement to a non-author institutional point of contact, such as a data access or ethics committee, and send this to us via return email. Please also include contact information for the third party organization, and please include the full citation of where the data can be found.

4. Please include captions for your Supporting Information files at the end of your manuscript, and update any in-text citations to match accordingly. Please see our Supporting Information guidelines for more information: http://journals.plos.org/plosone/s/supporting-information.

Additional Editor Comments (optional):

Well written study

Reviewers' comments:

Reviewer's Responses to Questions

**Comments to the Author**

1. Is the manuscript technically sound, and do the data support the conclusions?

The manuscript must describe a technically sound piece of scientific research with data that supports the conclusions. Experiments must have been conducted rigorously, with appropriate controls, replication, and sample sizes. The conclusions must be drawn appropriately based on the data presented.

Reviewer #1: Yes

Reviewer #2: Yes

**********

2. Has the statistical analysis been performed appropriately and rigorously?

Reviewer #1: Yes

Reviewer #2: Yes

**********

3. Have the authors made all data underlying the findings in their manuscript fully available?

The PLOS Data policy requires authors to make all data underlying the findings described in their manuscript fully available without restriction, with rare exception (please refer to the Data Availability Statement in the manuscript PDF file). The data should be provided as part of the manuscript or its supporting information, or deposited to a public repository. For example, in addition to summary statistics, the data points behind means, medians and variance measures should be available. If there are restrictions on publicly sharing data—e.g. participant privacy or use of data from a third party—those must be specified.

Reviewer #1: Yes

Reviewer #2: Yes

**********

4. Is the manuscript presented in an intelligible fashion and written in standard English?

PLOS ONE does not copyedit accepted manuscripts, so the language in submitted articles must be clear, correct, and unambiguous. Any typographical or grammatical errors should be corrected at revision, so please note any specific errors here.

Reviewer #1: Yes

Reviewer #2: Yes

**********

5. Review Comments to the Author

Please use the space provided to explain your answers to the questions above. You may also include additional comments for the author, including concerns about dual publication, research ethics, or publication ethics. (Please upload your review as an attachment if it exceeds 20,000 characters)

Reviewer #1: The investigators conducted a randomized, controlled, evaluator-blinded, multicentre superiority trial with a 1:1 allocation ratio among adults (≥18 years) with acute vestibular syndrome. The clinical effectiveness of online VR was compared with standard care consisting of written instructions.

The original sample size was targeted for about 320 participants. Due to slow accrual the entry was stopped at 184 an adaptive power recalculation was implemented. Under these assumptions, a total sample size 184 participants would be sufficient to detect a difference of 3.4 points in VSS-SF scores with 90% power at an alpha level of 5%. No attrition was incorporated in the updated power calculation as multiple imputation was used to handle missing data.

The statistical methods were sufficiently detailed. Both the primary outcome and the secondary outcomes were longitudinally evaluated using linear mixed models (LMM). The analysis was conducted on the per-protocol cohort and the number of participants at each time point is reported. The LMM was used to analyze outcomes as a function of time (four time points: baseline, 3, 6, and 12 weeks) and intervention group, with random effects for subject and site.

Although, statistically, in this multicenter randomized controlled trial, no differences were found in vestibular symptom reduction between internet-based vestibular rehabilitation (VR) and standardized written instructions among patients with acute vestibular syndrome (AVS), the paper was generally well written and the conclusions followed from the analyses performed. The strengths and limitations were noted. All the statistical design and analysis components were in place.

Reviewer #2: This randomized controlled trial compared Internet-based vestibular rehabilitation versus written instructions after acute vertigo in 184 participants and found that 184 randomised participants and reported that both groups improved at six weeks, with no significant between-group. Over 12 to 57 weeks, both groups showed reduced vestibular symptoms and improved disability, balance, and walking speed, again without significant between-group differences.

The caveat in this study was that the original sample size was that the sample size was reduced to 184 from the original 320 due to slower-than-anticipated recruitment.

Further, the results are mainly applicable to patients with vestibular neuritis and not other causes of acute vertigo. It should be emphasized that this form of vertigo spontaneously resolves in an overwhelming portion of affected patients due to its widely held viral etiology. This largely explains the similarity of results in both arms of the trial.

The Hawthorne effect exerted by the study team members who regularly checked on the patients can also explain the similarity of results in both arms.

Perhaps a more pragmatic approach could have made use of regular check by the participants' own primary care providers whose familiarity with them would have reduced observer bias.

This trial is important because, as the authors rightly pointed out, it improves access to vestibular rehabilitation among people who are not just remotely located from clinical caregivers but also unable to travel there as a result of the debilitating effects of their acute vertigo. It therefore offers hope to patients and some optimism to caregivers that a more individualized way of vestibular rehabilitation can be done without making the patients' condition worse, as long as they are internet savvy.

**********

6. PLOS authors have the option to publish the peer review history of their article (what does this mean?). If published, this will include your full peer review and any attached files.

If you choose “no”, your identity will remain anonymous but your review may still be made public.

**Do you want your identity to be public for this peer review?**  For information about this choice, including consent withdrawal, please see our Privacy Policy.

Reviewer #1: No

Reviewer #2: **Yes:**  Dr Jose M. Acuin

**********

# Editor report: Acceptance letter

**DOI:** 10.1371/journal.pone.0351092.r003

**Authors:** Jeyasakthy Saniasiaya

PONE-D-26-13474

PLOS One

Dear Dr. Salzer,

I'm pleased to inform you that your manuscript has been deemed suitable for publication in PLOS One. Congratulations! Your manuscript is now being handed over to our production team.

At this stage, our production department will prepare your paper for publication. This includes ensuring the following:

* All references, tables, and figures are properly cited

* All relevant supporting information is included in the manuscript submission,

* There are no issues that prevent the paper from being properly typeset

You will receive further instructions from the production team, including instructions on how to review your proof when it is ready. Please keep in mind that we are working through a large volume of accepted articles, so please give us a few days to review your paper and let you know the next and final steps.

Lastly, if your institution or institutions have a press office, please let them know about your upcoming paper now to help maximize its impact. If they'll be preparing press materials, please inform our press team within the next 48 hours. Your manuscript will remain under strict press embargo until 2 pm Eastern Time on the date of publication. For more information, please contact onepress@plos.org.

You will receive an invoice from PLOS for your publication fee after your manuscript has reached the completed accept phase. If you receive an email requesting payment before acceptance or for any other service, this may be a phishing scheme. Learn how to identify phishing emails and protect your accounts at https://explore.plos.org/phishing.

If we can help with anything else, please email us at customercare@plos.org.

Thank you for submitting your work to PLOS ONE and supporting open access.

Kind regards,

PLOS ONE Editorial Office Staff

on behalf of

Dr. Jeyasakthy Saniasiaya

Academic Editor

PLOS One
